# Identification of Pathogens and Biological Control of Wheat Fusarium Crown Rot in Xinjiang with *Pseudomonas aeruginosa* J-7

**DOI:** 10.3390/microorganisms14030627

**Published:** 2026-03-10

**Authors:** Cuicui Yan, Bin Zhang, Beibei Shi, Yejuan Du, Zheng Liu, Jiafeng Huang, Qinggui Lian

**Affiliations:** 1 Key Laboratory of Oasis Agricultural Pest Management and Plant Protection Resources Utilization, College of Agriculture, Shihezi University, Shihezi 832003, China; 19914347357@163.com (C.Y.); alenaweber76542@gmail.com (B.Z.); dyjagr@sina.com (Y.D.); lzh8200@126.com (Z.L.); 2 Key Laboratory of Cotton Biology, Genetics and Breeding, Northwest Inland Region, Ministry of Agriculture and Rural Affairs, Cotton Research Institute of Xinjiang Academy of Agricultural Sciences, Shihezi 832000, China; 3 Shaanxi Key Laboratory of Research and Utilization of Resource Plants on the Loess Plateau, College of Life Sciences, Yan’an University, Yan’an 716000, China; shibeibei@yau.edu.cn

**Keywords:** Fusarium crown rot, *Fusarium culmorum*, *Pseudomonas aeruginosa*, rhizosphere microbiome

## Abstract

Wheat Fusarium crown rot (FCR), predominantly caused by *Fusarium* species, is a devastating fungal disease that severely threatens global wheat production. In this study, we combined phytopathological assays, molecular techniques, and bioinformatic analyses to systematically identify the causal agents of FCR in Xinjiang and to screen for potential biocontrol bacteria. A total of 296 fungal isolates were obtained from 195 FCR samples, collected from Yumin County and Xinhe County. Morphological and phylogenetic analyses revealed that *Fusarium culmorum* was the predominant pathogen, accounting for 73.6% of the total isolates. To evaluate the resistance of local wheat cultivars, *F. culmorum* XN22-1, a highly virulent strain from Xinhe County, was inoculated to 30 wheat varieties. The results demonstrated that most cultivars lacked resistance to FCR, with the exception of three varieties—Xinchun 19, Xinchun 50, and Youpi 23, which showed a mid-resistance. Given the scarcity of resistant cultivars, we focused on biological control. To control FCR, *Pseudomonas aeruginosa* J-7, exhibiting broad-spectrum antagonistic activity, was successfully isolated from rhizosphere soil based on the analysis of healthy rhizosphere soil microbial diversity. Subsequently, pot experiments showed that *P. aeruginosa* J-7 could significantly reduce the disease incidence and lower the disease index of wheat FCR. Furthermore, whole-genome sequencing, in-plate metabolite analysis, and observation on inhibition of spores and mycelium revealed that *P. aeruginosa* J-7 mediates its biocontrol activity primarily through the production of phenazine and siderophores, which collectively inhibit conidial germination and cause structural damage to the mycelium. This study not only clarifies the composition of FCR pathogens in Xinjiang but also provides a promising biocontrol agent and new strategic insights for the management of wheat crown rot.

## 1. Introduction

Wheat is one of the world’s most important cereal crops and a staple food for billions of people, meeting critical global energy and nutritional needs [1,2]. While global demand for wheat continues to grow, its production is increasingly constrained by the cumulative effects of abiotic and biotic stresses [3]. Fusarium crown rot (FCR), a soil-borne disease caused by pathogenic *Fusarium* species, was first identified in Australia in 1951 and has since emerged as a pervasive global threat to wheat, resulting in substantial annual yield and quality reductions worldwide [4,5,6]. In Australia, FCR causes estimated annual losses of about $97 million in wheat and barley [7]. Similarly, in the U.S. Pacific Northwest, FCR may cause yield losses of up to 35% in winter wheat [8].

In China, FCR was first found in Henan (2012) and has spread to major wheat-growing regions with an increasing trend [9,10]. The disease caused substantial yield losses, reaching 30% in severely affected fields (e.g., Xuchang and Jiaozuo) and up to 38.0% in extreme cases [11]. Concurrently, the recent continuous expansion of wheat cultivation in Xinjiang has been accompanied by an increased incidence of pests and diseases, which pose a serious threat to production and adversely affect both quality and yield [12]. FCR is gradually becoming one of the most destructive diseases in wheat in Xinjiang.

In 2012, Li et al. first reported *F. pseudograminearum* as a causal agent of FCR in the Northwest wheat region [9]. Later, Yang et al. (2024) [13] identified a pathogen complex in Xinjiang’s spring wheat area consisting of *F. acuminatum*, *F. oxysporum*, and members of the *F. equiseti* species complex (FIESC). The pathogen composition of Fusarium crown rot in Xinjiang is complex, with multiple *Fusarium* species causing co-infection, which increases the difficulty of disease control.

Although resistant varieties are the most economical strategy for FCR control, fully resistant cultivars are unavailable. Only a few wheat lines with moderate resistance have been reported, most of which are old cultivars with low yield potential [12]. Owing to the lack of suitable resistant varieties, chemical control remains the main measure. However, both early and current fungicides only provide partial or short-term control, and their efficacy is limited at high disease severity; furthermore, they may cause chemical residue problems [14,15]. Therefore, sustainable alternatives such as biological control are urgently needed.

Compared to chemical control, biological control represents a more sustainable and eco-friendly alternative. Some studies have found that the composition and abundance of microbial communities in the rhizosphere are key determinants of plant health [16,17]. As a result, biological control (biocontrol) employing beneficial microorganisms has emerged as a prominent research area in sustainable disease control strategies [18,19,20,21,22]. Beneficial microorganisms mediate biocontrol through diverse mechanisms. Such as *Bacillus* spp., which could suppress *F. graminearum* via niche competition, antimicrobial lipopeptides, and host-induced berberine synthesis, reducing disease incidence by 53.9% [23]. The combined application of *B. amyloliquefaciens* T40 and *Bacillus pumilus* T208 could enhance rice resistance to *F. oxysporum* by modulating the rhizosphere microbiome and producing cyclic lipopeptides, such as surfactin and iturin, which directly inhibit the pathogen and induce systemic immunity [24,25]. Additionally, *Chaetomium globosum* G3 reduces *F. verticillioides*-induced maize seedling blight incidence from 81.5% to 37.6% through iron competition, enzymatic antagonism, and inhibition of fumonisin biosynthesis [26].

Although numerous studies have focused on developing biocontrol strains against FCR, their field performance has often been inconsistent. Several biocontrol agents have shown antagonistic activity against *F. pseudograminearum* in laboratory tests: O’Sullivan et al. identified two *Streptomyces strains* (MH71, MH243) with strong inhibitory effects in dual-culture and seedling assays, but their control efficacy remained low under greenhouse conditions [27]; Liu et al. found that *Phanerochaete chrysosporium* inhibits the pathogen in vitro, yet its limited efficacy in pot trials indicates poor field applicability [28]. Collectively, these studies show that while many biocontrol agents work well in laboratory settings, their reduced efficacy in simulated (greenhouse/pot) environments is the main reason they fail to translate to practical field use. Although *F. pseudograminearum* is widely recognized as the primary causal agent of wheat crown rot, systematic and in-depth studies on *F. culmorum* are still severely insufficient, leaving a critical knowledge gap. This gap constitutes the key scientific rationale and highlights the novelty of the present study.

Based on the above research gaps, this study combined phytopathological assays, molecular techniques, and bioinformatic analyses to systematically identify the causal agents of FCR in Xinjiang and to screen for potential biocontrol bacteria. Our preliminary results indicate that *F. culmorum* is the dominant pathogen of wheat FCR in Xinjiang, and *P. aeruginosa* shows potential in significantly reducing the disease index of wheat FCR by damaging pathogenic mycelium and inhibiting conidial germination. In addition, many previous studies have verified that *P. aeruginosa* strains possess remarkable biocontrol properties against diverse plant fungal diseases, providing reliable theoretical and experimental support for its selection in this study.

## 2. Materials and Methods

### 2.1. Disease Investigation and Pathogen Isolation and Identification

Field surveys were conducted at different growth stages (seedling and adult growth stages) across two locations (Yumin and Xinhe). To avoid edge effects, sampling commenced 5 m from the field border. We randomly established five points per field, assessing 30 plants per point. Disease incidence was visually estimated as the percentage of affected plants per plot, while severity was scored on a 0–5 scale following Martin’s method [29] (Supplementary Table S1). The incidence rate was calculated as follows: (Number of diseased plants/Total number of inoculated plants) × 100. The disease index (DI) = [∑(Number of plants in each severity grade × Severity grade)/(Total number of plants investigated × Highest severity grade)] × 100.

The collected samples were subjected to fungal isolation and purification via the tissue separation method, following the protocol outlined in ‘*Experiences with the Isolation of Plant Pathogenic Fungi*’ [30]. A total of 296 *Fusarium* isolates were obtained from wheat plants showing typical crown rot symptoms. The resulting isolates were preliminarily categorized according to the ‘*Fungal Identification Manual*’ assessing their colony morphology on potato dextrose agar (PDA) and conidial structures on carnation leaf agar (CLA). For pathogenicity assessment, representative 3 isolates from each morphological category were selected and inoculated into plants using a stem base drip method, as detailed by Mitter et al. [31] Subsequently, to satisfy Koch’s postulates, the pathogen was re-isolated from the symptomatic plant tissues and confirmed to be morphologically identical to the original inoculated strain.

The pathogenic strains were further morphologically identified by culture morphology on 8 madia (PDA, PSA, OA, VBC, CLA, CA, SNA, and MGA). All isolates were routinely cultured on these media for preliminary morphological identification, according to the *Fusarium* Laboratory Manual, Guidelines for the Identification of Common *Fusarium* Species, and Leslie & Summerell [32]. Then the genomic DNA of the pathogenic strain was extracted using the *Biospin* fungal genomic DNA extraction kit (TransGen Biotech, Beijing, China). The ITS region and the translation elongation factor 1-α (*tef1-α*) gene (Table S4) were amplified by with primers ITS1/ITS4 and EF1-728F/EF1-986R, respectively. The reaction system and amplification conditions were shown in Tables S2 and S3. The PCR products were visualized on 1% agarose gel and sequenced by Sanger method. The sequences were assembled and aligned, and phylogenetic analysis was performed using MEGA 7.0 based on concatenated *tef1-α* and ITS sequences. The phylogenetic tree was constructed by the Neighbor-Joining (NJ) method with 1000 bootstrap replicates to evaluate the reliability of the tree topology. The mating-type of 24 *Fusarium* class I isolates was detected via PCR amplification of mating-type idiomorph genes. Genomic DNA of the isolates (extracted via the *Biospin* fungal genomic DNA extraction kit, TransGen Biotech, Beijing, China) was used as the template, and the specific primers for mating-type amplification were MAT1-1F/MAT1-1R and MAT1-2F/MAT1-2R (Table S4). The PCR reaction system (25 μL) was composed of: 12.5 μL 2 × Taq PCR Master Mix (Tiangen, Beijing, China), 1 μL of each forward and reverse primer (10 μmol/L), 2 μL genomic DNA template (50 ng/μL), and 8.5 μL sterile double-distilled water. The PCR amplification program was set as: pre-denaturation at 94 °C for 5 min; 35 cycles of denaturation at 94 °C for 30 s, annealing at 58 °C for 30 s, extension at 72 °C for 45 s; final extension at 72 °C for 10 min, and preservation at 4 °C. The PCR products were separated by 1.0% agarose gel electrophoresis (120 V, 30 min), stained with ethidium bromide, and visualized under a gel imaging system (Bio-Rad, Hercules, CA, USA). The mating-type of each isolate was determined according to the presence of specific amplification bands: the appearance of a target band with MAT1-1F/MAT1-1R primers indicated the MAT1-1 mating-type, while a target band with MAT1-2F/MAT1-2R primers indicated the MAT1-2 mating-type. Three technical replicates were set for each sample to ensure the accuracy of detection results.

### 2.2. Resistance Evaluation of Wheat Varieties

The *F. culmorum* strain XN22-1 was selected for pathogenicity testing. A total of 30 wheat (Thirty wheat germplasm resources were involved in this study, with their detailed information (e.g., variety name, origin, and basic traits) provided in Table S9 of the Supplementary Materials) germplasm resources were evaluated. Sterilized nutrient soil and vermiculite were mixed at a ratio of 3:1, thoroughly mixed and filled into 5 × 5 × 5 cm black square pots. Wheat seeds with similar bud lengths were selected for sowing, with a sowing depth of approximately 1.5–2.5 cm, and 10 seeds were sown in each black square pot. The *F. culmorum* strain XN22-1 was inoculated into CMC medium and cultured with shaking at 28 °C for 7 days. The culture was filtered through 3 layers of sterile gauze to remove mycelial fragments, and the spore concentration was adjusted to 1 × 10^6^ colony-forming units conidia/mL. The experiment was set with 3 biological replicates and 1 blank control group (inoculated with sterile distilled water instead of fungal inoculum). All experimental plants were maintained in a greenhouse at 24 °C with a photoperiod of 12 h of light/12 h of dark. The disease index was evaluated 50 days after inoculation.

The disease index (DI) was calculated, and the resistance of each wheat germplasm to Fusarium crown rot (FCR) was evaluated based on the average DI, according to the criteria detailed in Table S5.

### 2.3. Soil Sampling and Pot Experiment Setup

The purpose of this pot experiment was to initially verify that beneficial microorganisms in healthy soil (collected from disease-occurring areas) can resist pathogens and reduce the disease index. The experimental soil was natural saline-alkali clay soil with a clay texture and a pH of 8.4. Specifically, we compared wheat disease indices after inoculating the same pathogen in healthy and diseased soils, providing preliminary evidence for the disease-suppressive effect of beneficial microorganisms in healthy soil. Rhizosphere soil samples were collected from Xinhe County (a disease-occurring area): diseased samples from five wheat fields under continuous monoculture for at least three years (prone to *F. culmorum*-induced wheat diseases), and healthy samples from five newly planted or reclaimed wheat fields (no obvious wheat diseases). For the pot experiment, a portion of each soil type (healthy and diseased) was sterilized by autoclaving at 121 °C for 15 min (to eliminate all microorganisms, including beneficial ones), and the remainder was used as non-sterilized natural soil (to retain indigenous microorganisms). Seeds of the local prevalent cultivar Xindong 22 were surface-sterilized with 10% NaOCl for 30 min, rinsed thoroughly with sterile water, soaked for 12 h, and germinated in vermiculite. Each experimental unit was a 5 × 5 × 5 cm black square potfilled with 50 g of soil, with 10 seedlings per pot, with seedlings planted at 1.5–2.5 cm depth in four soil types: non-sterilized healthy soil, sterilized healthy soil, non-sterilized diseased soil, and sterilized diseased soil. The experiment included three replicates per soil treatment and one blank control group (inoculated with sterile distilled water instead of fungal spore suspension, with the same soil types and planting conditions as the treatments) to exclude interference from other factors. At the two-leaf stage, all treatment seedlings were given a single inoculation with 1 × 10^6^ conidia/mL *F. culmorum* XN22-1 spore suspension (uniform inoculation conditions for all treatments) and maintained in a greenhouse at 24 °C with a 12 h light/12 h dark photoperiod. During the experiment, sterile distilled water was irrigated every 2 days to maintain soil moisture at 60 ± 5% (avoiding waterlogging), ensuring normal wheat growth, stable *F. culmorum* infection, and normal activity of indigenous microorganisms in non-sterilized soil. Disease index was assessed 14 days post-inoculation to compare disease severity among treatments and initially confirm the disease-resisting effect of beneficial microorganisms in healthy soil.

### 2.4. Microbiome Sequencing and Bioinformatic Analysis

Subsamples of healthy and diseased rhizosphere soils (three replicates each) using the E.Z.N.A.^®^ Soil DNA Kit (Omega Bio-tek, Inc., Norcross, GA, USA). The DNA samples were shipped on dry ice to Majorbio Bio-pharm Technology Co. Ltd. (Shanghai, China) for high-throughput sequencing on an Illumina Nextseq2000 platform (Illumina, San Diego, CA, USA). The V3–V4 hypervariable region of the bacterial 16S rRNA gene was amplified using primers (Supplementary Table S3) 338F/806R (The PCR reaction mixture including 4 μL 5× Fast Pfu buffer, 2 μL 2.5 mM dNTPs, 0.8 μL each primer (5 μM), 0.4 μL Fast Pfu polymerase, 10 ng of template DNA, and ddH_2_O to a final volume of 20 µL. PCR amplification cycling conditions were as follows: initial denaturation at 95 °C for 3 min, followed by 27 cycles of denaturing at 95 °C for 30 s, annealing at 55 °C for 30 s and extension at 72 °C for 45 s, and single extension at 72 °C for 10 min, and end at 4 °C), and the ITS1 region of fungi was amplified with primers ITS1F/ITS2R (The PCR reaction mixture including 4 μL 5× Fast Pfu buffer, 2 μL 2.5 mM dNTPs, 0.8 μL each primer (5 μM), 0.4 μL Fast Pfu polymerase, 10 ng of template DNA, and ddH_2_O to a final volume of 20 µL. PCR amplification cycling conditions were as follows: initial denaturation at 95 °C for 3 min, followed by 27 cycles of denaturing at 95 °C for 30 s, annealing at 55 °C for 30 s and extension at 72 °C for 45 s, and single extension at 72 °C for 10 min, and end at 4 °C). Raw sequences were processed in QIIME 1.91, including quality filtering, chimera removal, and clustering into operational taxonomic units (OTUs) at 97% similarity using USEARCH (v10.0). OTUs representing less than 0.005% of total reads were discarded. All samples were rarefied to an equal sequencing depth, and taxonomy was assigned against the SILVA database. Community composition was visualized using bar plots of dominant taxa (GraphPad Prism 10). Alpha diversity (Shannon, Simpson, Chao1, and ACE indices) and beta diversity (PCoA and NMDS based on Bray–Curtis distance) were analyzed using the vegan package in R (v4.2.3). Differential taxa between healthy and diseased rhizospheres were identified by LEfSe analysis on the Majorbio cloud platform, using a Kruskal–Wallis test with *p* < 0.05 for significance testing and an LDA score threshold of ≥2.0 for effect size filtering.

### 2.5. Screening of Biocontrol Bacteria and Determination of Broad-Spectrum Antagonism

Bacteria were isolated from healthy rhizosphere soil samples using the serial dilution method. Specifically, 1 g of soil was suspended in 100 mL of sterile water and shaken at 150 rpm for 30 min. The suspension was serially diluted in sterile water to 10^−6^, and 200 μL from appropriate dilutions was spread on LB agar plates (three replicates per dilution). After incubation at 30 °C for 24 h, distinct colonies were selected, repeatedly streaked for purity, and stored as glycerol stocks at −80 °C.

Antagonistic activity was assessed using a dual-culture assay. A mycelial plug of *F. culmorum* XN22-1 was placed at the center of a PDA plate, and bacterial isolates were spot-inoculated at four equidistant points, 2 cm from the pathogen. Plates were incubated at 28 °C for 7–10 days, and the inhibition zone width was measured. The relative inhibition rate was calculated as follows:

Inhibition rate (%) = [(Control colony diameter − Treatment colony diameter)/Control colony diameter] × 100. The broad-spectrum antagonistic activity of the most potent isolate, J-7, was further evaluated against additional wheat FCR pathogens (*F. pseudograminearum*, *F. graminearum*, *B. sorokiniana*) and other soil-borne pathogens (*Verticillium dahliae*, *Rhizoctonia solani*, *Cytospora chrysosperma*, *Macrophomina phaseolina*, *Trichothecium roseum*, *Valsa pyri*) using the same dual-culture method.

### 2.6. Molecular Identification of Isolate J-7

Isolate J-7 was characterized morphologically on LB agar and through Gram staining. Genomic DNA of *P. aeruginosa* J-7 was extracted using the E.Z.N.A.^®^ Bacterial DNA Kit (Omega Bio-tek, Norcross, GA, USA). The 16S rRNA gene was amplified by PCR with universal primers 27F (5′-AGAGTTTGATCCTGGCTCAG-3′) and 1492R (5′-GGTTACCTTGTTACGACTT-3′). The 25 μL PCR mixture contained 12.5 μL of 2× Taq PCR MasterMix, 1 μL of 10 μM forward primer, 1 μL of 10 μM reverse primer, 1 μL of template DNA, and 9.5 μL of sterile ddH_2_O. Thermal cycling conditions were as follows: 95 °C for 5 min, followed by 30 cycles of 95 °C for 30 s, 55 °C for 30 s, and 72 °C for 90 s, with a final extension at 72 °C for 10 min. Whole-genome sequencing was performed using the Illumina platform. The whole-genome sequencing data of *P. aeruginosa* J-7 were deposited in the Genome Sequence Archive (GSA) under the accession number CRA037583.The phylogenetic tree of strain J-7 was constructed in MEGA 7.0 using the neighbor-joining method, and the tree topology was verified by bootstrap analysis with 1000 replicate bootstrap values to ensure its reliability.

### 2.7. Biocontrol and Growth Promotion Effects of P. aeruginosa J-7

Wheat seeds (Xindong 48) were used. Seeds were first surface-sterilized with 10% NaOCl for 30 min, rinsed thoroughly with sterile water, soaked for 12 h, and pre-germinated in sterile vermiculite. After germination, only seedlings without visible fungal growth or abnormalities were selected for subsequent experiments to ensure that the seeds/seedlings were not infected by other plant pathogens. Each replicate contained 10 wheat seedlings, each treatment included three biological replicates, and the entire experiment was repeated three times independently. Three treatments were established, each with three replicates: Wheat seedlings at the two-leaf stage were treated as follows:

First, the preparation procedures of bacterial inoculum and fungal spore suspension were clarified: The *P. aeruginosa* J-7 bacterial inoculum was prepared as a pre-inoculum: J-7 strain was inoculated into LB liquid medium and cultured at 30 °C with shaking at 150 rpm for 24 h, then adjusted to OD_600_ = 1(1 × 10^8^ CFU/mL) with sterile water to obtain the bacterial suspension used in the experiment. The *F. culmorum* XN22-1 spore suspension (1 × 10^6^ conidia/mL) was obtained by inoculating XN22-1 strain into CMC medium, culturing with shaking at 28 °C for 7 days, filtering through 3 layers of sterile gauze to remove mycelial fragments, and adjusting the spore concentration with sterile water using a hemocytometer.

Treatment 1 (J-7 + *F. culmorum*) received 5 mL of J-7 bacterial suspension (OD_600_ = 1), followed two days later by 2 mL of XN22-1 spore suspension (1 × 10^6^ conidia/mL). Treatment 2 (*F. culmorum*) received 5 mL of sterile water, followed by the same spore inoculation as Treatment 1. Treatment 3 (CK control) received sterile water in both applications.

All experimental plants were maintained under controlled conditions at 24 °C with a 12 h light/12 h dark photoperiod; sterile distilled water was irrigated every 2 days to keep the soil moisture at 60 ± 5% (avoiding waterlogging). The trial duration was 28 days (consistent with the growth promotion assay). Disease incidence and index were assessed 21 days post-inoculation.

Growth promotion assay: To assess plant growth promotion, 10 wheat seedlings were used per replicate, with three biological replicates, and the experiment was repeated three times independently. wheat plants at the two-leaf stage (selected by the same pre-germination and screening method mentioned above) were treated with 2 mL of *P. aeruginosa* J-7 bacterial suspension (OD_600_ = 1) via root drenching. Control plants received sterile water. All plants were maintained under the same controlled conditions (24 °C, 12 h light/12 h dark photoperiod, regular irrigation) for 28 days, after which growth parameters including plant height, fresh weight, and dry weight were measured and recorded.

### 2.8. Whole Genome Sequencing and Analysis

The whole genome of *P. aeruginosa* J-7 was sequenced on the PacBio Sequel II platform (Shanghai Meiji Biomedical Technology Co., Ltd., Shanghai, China). The subsequent bioinformatic analysis involved: quality control and filtering of raw data with fastp v0.20.0; genome assembly using third-generation software with quality assessed by BUSCO v4.5.5; and comprehensive genomic component annotation using Prodigal [33] v2.6.3/GeneMarkS [34] v4.3 (genes), TRF v4.09.1/RepeatMasker v4.1.5 (repeats), Minced v0.2.0 (CRISPR arrays), barrnap v0.9 (https://github.com/tseemann/barrnap/, accessed on 24 February 2026) /tRNAscan-SE [35] v2.0.12/Infernal v1.1.5 (non-coding RNAs), and Phigaro v2.4.0 (prophages). Functionally, proteins were annotated against NR, Swiss-Prot, and KEGG via Diamond v0.8.35/BLAST+ v2.3.0; secondary metabolite gene clusters were predicted with antiSMASH v7.0.0; and virulence/host-interaction genes were identified using VFDB (http://www.mgc.ac.cn/VFs/main.htm, accessed on 24 February 2026). and PHI v5.0 (http://www.phi-base.org/, accessed on 24 February 2026). databases to elucidate potential antifungal mechanisms.

### 2.9. Detection of Growth-Promoting Factors and Cell Wall-Degrading Enzyme Production Capacity of the Biocontrol Bacterium

Bacterial plugs (6 mm) of *P. aeruginosa* J-7 were inoculated onto chrome azurol S (CAS) agar for siderophore detection, as well as organic and inorganic phosphorus media for phosphate solubilization assays. Plates were incubated statically at 28 °C for 3 days. For all plate-based assays, the potency index (PI) was quantified as the core evaluation index, calculated by the formula: PI = Diameter of the characteristic halo/clear zone (D, cm)/Diameter of the bacterial colony (d, cm). Orange halo zones on CAS agar indicated siderophore production, and the siderophore production capacity was quantified by the PI value of the orange halo zone to the colony; clear zones on phosphorus media indicated phosphate solubilization capacity, and the phosphate solubilization ability was quantified by the PI value of the phosphorus solubilization clear zone to the colony. LB medium plugs served as the control. Each assay was performed in triplicate.

To assess hydrolytic enzyme production, *P. aeruginosa* J-7 was inoculated onto carboxymethyl cellulose (CMC) medium for cellulase, skim milk agar for protease, and starch agar for amylase activity. After 3 days of incubation at 28 °C (LB medium plugs served as the control), CMC plates were stained with Congo red (1%), skim milk plates were directly observed for clearance zones, and starch plates were treated with Lugol’s iodine solution. The appearance of clear hydrolysis zones around colonies indicated positive enzymatic activity, and the activity of cellulase, protease and amylase was separately quantified by the PI value of the corresponding hydrolysis clear zone to the colony for each enzyme detection plate. All experiments included controls and were repeated three times.

### 2.10. Study on Inhibition of Pathogen Spores and Mycelium

Spore germination assay: a 1:1 mixture of *P. aeruginosa* J-7 bacterial suspension (OD_600_ = 1) and *F. culmorum* XN22-1 conidial suspension was prepared (1 × 10^6^ conidia/mL), while the control received sterile water instead of bacterial suspension. The mixtures were incubated at 25 °C, and conidial germination was assessed at 1 h intervals under a light microscope. Germination was defined as the emergence of a germ tube exceeding two-thirds of the conidium length. The percentage of germinated conidia was recorded for each treatment. Mycelial inhibition assay: Mycelial plugs from the inhibition zone in dual-culture assays with XN22-1 were collected at various time points, stained with 0.4% trypan blue solution, and examined microscopically for structural abnormalities. The trypan blue staining solution (0.4%) was prepared in phosphate-buffered saline (PBS) and stored at 4 °C prior to use.

### 2.11. Quantification and Statistical Analysis

All statistical analyses were performed using SPSS software (version 19.0), and a *p* value < 0.05 was considered statistically significant. Data visualization (histograms) was completed using GraphPad Prism software (version 10.0), and all data are presented as mean ± SEM (*n* = 3 independent biological replicates).

Biological data (including wheat growth parameters such as plant height, fresh weight, dry weight, and disease incidence/index) were subjected to two-tailed Student’s *t*-test, as these data conformed to the normal distribution and homogeneity of variance, which is the applicable condition for Student’s *t*-test.

Microbiological data (including alpha diversity indices such as Chao1, Shannon, and Simpson, as well as intergroup differences in microbial community composition) were analyzed using Wilcoxon rank-sum test, which is suitable for non-normally distributed data or data that do not meet the assumptions of parametric tests.

Bioinformatic analysis was conducted on the Majorbio Cloud platform. Taxonomic annotation of bacterial 16S rRNA gene sequences and fungal ITS sequences was performed against the SILVA 16S rRNA database (v138) and UNITE database (v8.2), respectively. Principal coordinate analysis (PCoA) based on Bray–Curtis distance was used to evaluate the similarity of microbial community structure among samples, and PERMANOVA test was further used to verify significant differences in community structure between groups. LEfSe analysis (LDA score > 2, *p* < 0.05) was employed to identify significantly differential microbial taxa from phylum to genus level among different treatment groups.

## 3. Results

### 3.1. The Incidence of Wheat FCR in Xinjiang Is Increasing Year by Year

**Figure 1:**
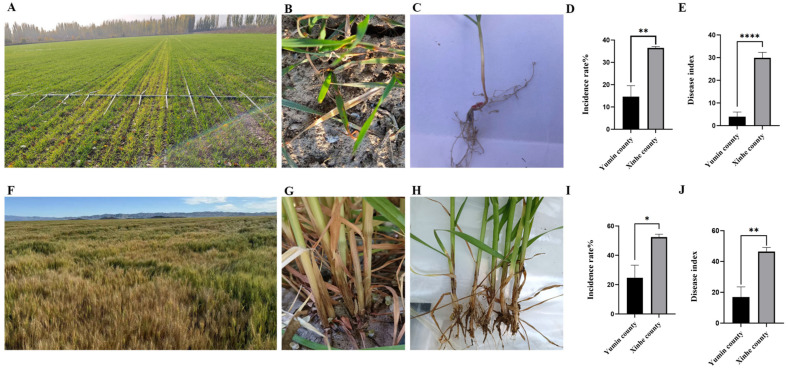
Symptoms and disease indices of wheat Fusarium crown rot. (**A**–**C**) Field symptoms of FCR at wheat seedling stage. (**D**,**E**) Field disease incidence and index at seedling stage. (**F**–**H**) Field symptoms of FCR at wheat adult stage. (**I**,**J**) Field disease incidence and index at adult stage.Data are mean ± SEM (*n* = 3 independent biological replicates). * *p* < 0.05, ** *p* < 0.01, **** *p* < 0.0001 (two-tailed Student’s *t*-test).

Field surveys in Yumin (Tacheng) and Xinhe (Aksu) counties during 2022–2023 showed that wheat Fusarium crown rot (FCR) occurred at both seedling and adult stages. At the seedling stage, infected plants exhibited leaf yellowing and yellow-brown to dark brown necrotic lesions on the stem base, with obvious chlorotic patches in severely diseased fields (Figure 1A–C). At the adult stage, dark brown necrosis and pink mycelial mats appeared on the stem base; spikes showed chlorosis and whitening, and severely infected plants were stunted with premature senescence and sterile shriveled white spikes (Figure 1F–H).

Disease incidence and severity were significantly higher in Xinhe County than in Yumin County. In Yumin, the average seedling incidence was 14.60%, with a disease index of 3.92, increasing to 36.48% and 29.90, respectively, at the adult growth stage. In contrast, Xinhe County recorded 24.60% incidence and a disease index of 16.88 at the seedling stage, rising to 52.40% and 46.44 at the adult growth stage (Figure 1D,E,I,J). These results indicate that FCR severity escalates from seedling to adult growth stages and is more widespread and severe in Xinhe County.

### 3.2. F. culmorum Is the Dominant Pathogen of Wheat FCR in Xinjiang

**Figure 2:**
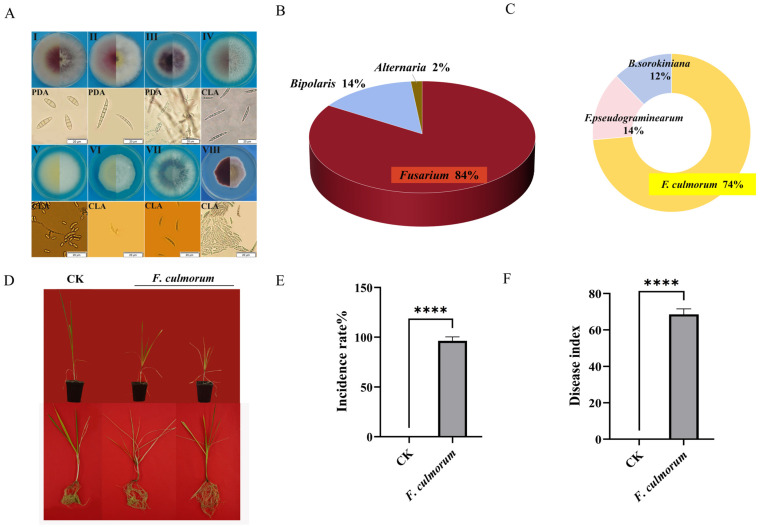
Pathogen isolation and pathogenicity identification of wheat stem rot. Note: I–VIII is the colony morphology of eight types of fungi isolated from diseased samples on PDA. (**A**) The culture status and spore morphology of eight *Fusarium* species on PDA and CLA. (**B**) Preliminary statistical pie chart of pathogen isolation frequency. (**C**) Pie chart of three pathogenic bacteria with pathogenicity. (**D**) The growth and incidence of wheat four weeks after inoculation with *Fusarium* II (CK (control check) = blank control group, wheat seedlings inoculated with sterile distilled water instead of *Fusarium* spore suspension under the same culture conditions). (**E**) The average incidence of wheat after 4 weeks of inoculation with *Fusarium* II. (**F**) Disease index of wheat 4 weeks after inoculation with *Fusarium* II. Disease index of wheat 4 weeks after inoculation with *Fusarium* II. Data are shown as mean ± SEM (*n* = 3 independent biological replicates). Statistical significance was determined by two-tailed Student’s *t*-test (**** *p* < 0.0001).

A total of 296 fungal isolates were obtained from 195 (Table S7) diseased wheat samples collected across different regions and growth stages through single-spore purification. Preliminary classification based on colony morphology and conidial characteristics on PDA after 7 days revealed that *Fusarium* spp. predominated (83.8%), followed by *Bipolaris* spp. (14.5%) and *Alternaria* spp. (1.7%) (Figure 2A), indicating *Fusarium* as the primary genus associated with FCR in Xinjiang (Figure 2B).

The 195 *Fusarium* isolates were classified into eight morphotypes according to their cultural characteristics on PDA and conidial morphology on PDA or CLA (Figure 2A). Pathogenicity tests using two representative isolates from each category demonstrated that only isolates from *Fusarium* I and *Fusarium* II induced typical FCR symptoms. Category I isolates caused disease indices 68.55 (Figure 2D–F), while *Fusarium* II isolates resulted in indices 76.35 (Figure S1A–C). *Fusarium* III isolates induced only limited heart leaf wilting without characteristic FCR symptoms, and *Fusarium* IV–VIII were non-pathogenic. All *Bipolaris* isolates (classified as *Bipolaris* I) were also pathogenic, with a mean disease index of 33.84 (Figure S1D–F).

**Figure 3:**
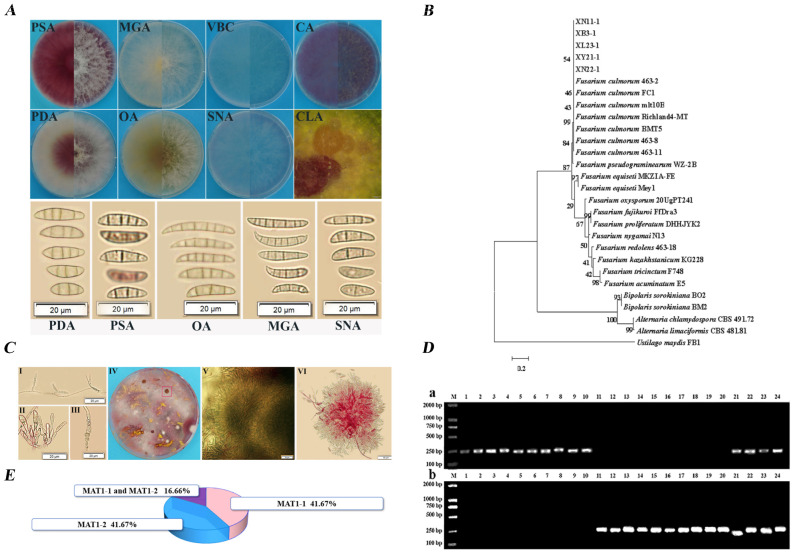
Morphological identification, phylogenetic tree analysis and mating type identification of *Fusarium* I. (**A**) The culture characteristics and conidial morphology of the representative strain XN22-1 of *Fusarium* on 8 different media. (**B**) A phylogenetic tree based on *tef1-α* gene and ITS region nucleotide sequence was constructed. (**C**) Microscopic characteristics of the representative strain XN22-1 of *Fusarium* I. Note: I, II: Conidiophores formed on PDA; III: sentchlamydospores cultured on PDA; IV: The red framed part on the petri dish is the myxosporium formed by *Fusarium* I on PDA; V: Microstructure of myxosporium formed by *Fusarium* I on PDA; VI: Conidiophores produced on PSA. (**D**) Mating type detection of 24 strains of *Fusarium* I. Note: (**a**) *Fusarium* I isolated from wheat at adult stage; (**b**) *Fusarium* I from wheat at seedling stage. (**E**) Mating type proportion pie chart of 24 strains of *Fusarium* I.

Further morphological and molecular characterization confirmed the identity of the pathogenic groups. Representative isolate XN22-1 (*Fusarium* I) was cultured on eight different media (PDA, PSA, MGA, OA, VBC, SNA, CA, CLA) and exhibited morphological features consistent with *F. culmorum* (Figure 3A,D). Molecular analysis based on concatenated *tef1-α* and ITS sequences placed all five representative *Fusarium* isolates within a clade containing reference *F. culmorum* strains (Figure 3B). Consistent with the morphological identification, molecular analysis confirmed that *Fusarium* I isolate XN22-1 was *Fusarium culmorum*. Mating-type analysis of 24 *Fusarium* isolates showed that 10 were MAT1-1, 10 were MAT1-2, and 4 yielded positive PCR results for both MAT1-1 and MAT1-2 idiomorphs (Figure 3C,E).

Similarly, *Fusarium* II and *Bipolaris* I were identified as *Fusarium pseudograminearum* (Figure S2A,B) and *Bipolaris sorokiniana* (Figure S2C,D, respectively, through morphological and multi-locus phylogenetic analysis. Final species distribution analysis confirmed *F. culmorum* as the dominant pathogen, comprising 73.6% (78 isolates) of the pathogenic isolates, followed by *F. pseudograminearum* (14.1%, 15 isolates) and *B. sorokiniana* (12.3%, 13 isolates) (Figure 2C and Table S8). These results establish *F. culmorum* as the principal causative agent of wheat FCR in Xinjiang.

### 3.3. Xinjiang Lacks Wheat Varieties Resistant to FCR

Pathogenicity screening of 30 wheat varieties inoculated with *F. culmorum* XN22-1 revealed severe susceptibility to FCR across most cultivars (Table S9). Only three varieties—Xinchun 19, Xinchun 50, and Youpi 23—exhibited mid-resistance, with mean disease indices of 22.50, 25.00, and 30.00, respectively. One variety, Huachangmai 26, was susceptible (disease index 40.00), while the remaining 26 varieties were highly susceptible, all showing disease indices exceeding 41.25. These results demonstrate that among the tested wheat germplasms, only 10% displayed moderate resistance to *F. culmorum*, whereas the majority (86.7%) were susceptible or highly susceptible. This clear lack of resistant varieties underscores the urgent need to incorporate FCR resistance into wheat breeding programs targeting Xinjiang and similar agro-ecological regions.

### 3.4. Soil Microorganisms Reduce the Disease Index of Wheat FCR

**Figure 4:**
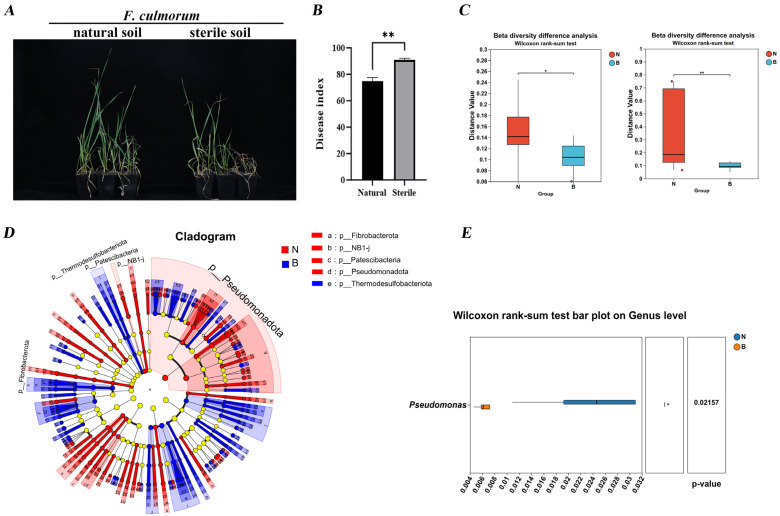
Analysis of soil microbial diversity. (**A**) Wheat growth and disease symptoms at 14 days after *F. culmorum* inoculation. (**B**) Wheat disease index at 14 days post inoculation. (**C**) Bacterial (left) and fungal (right) beta diversity analysis. (**D**) Bacterial LEfSe multi-level species cladogram. (**E**) Two-group comparison box plots. Data are mean ± SEM (*n* = 3 independent biological replicates). * *p* < 0.05, ** *p* < 0.01 (Student’s *t*-test for (**B**,**C**); Wilcoxon rank-sum test for (**E**)).

To identify biocontrol agents with potential for local application, we compared the suppressive capacity of rhizosphere soils from healthy and FCR-diseased wheat plants. In pot experiments using the prevalent local cultivar Xindong 22, plants grown in non-sterilized healthy rhizosphere soil showed significantly reduced FCR symptoms compared to those in sterilized soil after inoculation with *F. culmorum* XN22-1 (1 × 10^6^ conidia/mL) (Figure 4A). Quantitative assessment revealed that the native soil microbiome reduced the disease index from 90.67 in sterilized soil to 74.67 in non-sterilized soil (Figure 4B), demonstrating that indigenous microorganisms in healthy rhizosphere soil significantly suppress FCR development.

### 3.5. Pseudomonas Is Significantly Enriched in the Rhizosphere Soil of Healthy Plants

To further characterize the rhizosphere microbiome, we performed high-throughput sequencing of bacterial and fungal communities from five healthy (N) and five diseased (B) wheat rhizosphere soil samples, each with three replicates. A total of 1,531,459 high-quality bacterial sequences (641,994,582 bp, average length 419 bp) were obtained, clustered into 8845 OTUs, and annotated into 45 phyla and 1230 genera. For fungi, 2,032,345 high-quality sequences (491,306,870 bp, average length 241 bp) yielded 2341 OTUs, representing 17 phyla and 491 genera (Figure S3A). Rarefaction curves indicated sufficient sequencing depth to capture microbial diversity (Figure S3A).

At the phylum level (Figure S3B), the bacterial community was dominated by Pseudomonadota (31.87%), Bacteroidota (15.74%), and Bacillota (9.34%), whereas the fungal community was primarily composed of Ascomycota (61.75%), Olpidiomycota (18.94%), and Mortierellomycota (10.91%). Beta diversity analysis using PLS-DA revealed clear separation between the N and B groups (Figure S3C), indicating distinct bacterial and fungal community structures between healthy and diseased rhizospheres. Permutation testing confirmed significant differences in microbial community composition between the two groups (Figure 4C). Venn analysis at the phylum level (Figure S3D,E) showed that Pseudomonadota was the most abundant bacterial phylum in both groups (30.06%). At the genus level (Figure S3F), *Pseudomonas* under Pseudomonadota was significantly more abundant in the N group, whereas *Fusarium* was enriched in the B group. LEFSe analysis (LDA > 4, *p* < 0.05) identified Pseudomonadota as a significantly enriched biomarker in the N group (Figure 4D). Differential analysis at the genus level further confirmed that *Pseudomonas* was significantly more abundant in healthy rhizosphere soils (Figure 4E). These results collectively demonstrate that *Pseudomonas* is notably enriched in the rhizosphere of healthy wheat plants.

### 3.6. P. aeruginosa Isolate J-7 Exhibits Broad-Spectrum Antagonistic Activity Against Plant Pathogens

**Figure 5:**
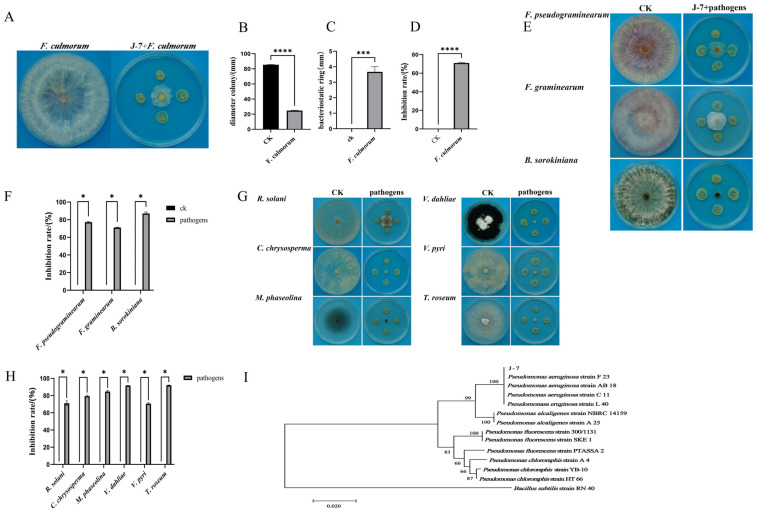
Inhibitory effects and identification of isolate J-7. (**A**) Dual-culture plate showing inhibition of *F. culmorum* mycelial growth by isolate J-7. (**B**) Colony diameter of *F. culmorum* in the presence of isolate J-7. (**C**) Measurement of the inhibition zone against *F. culmorum*. (**D**) Inhibition rate of isolate J-7 against *F. culmorum* mycelial growth. (**E**) Dual-culture plates showing inhibition of other wheat FCR pathogens by isolate J-7. (**F**) Inhibition rates of isolate J-7 against other wheat FCR pathogens. (**G**) Dual-culture plates showing inhibition of soil-borne pathogens by isolate J-7. (**H**) Inhibition rates of isolate J-7 against soil-borne pathogens. (**I**) Phylogenetic tree of isolate J-7 based on 16S rDNA sequence. Data are shown as mean ± SEM (*n* = 3 independent biological replicates). Statistical significance was determined by two-tailed Student’s *t*-test (* *p* < 0.05, *** *p* < 0.001, **** *p* < 0.0001).

Fifty-four bacterial isolates were obtained from healthy rhizosphere soils. To identify the most potent biocontrol candidate, all isolates were initially screened for antagonistic activity against *F. culmorum* XN22-1 using a dual-culture assay. Among them, isolate J-7 exhibited the most pronounced inhibitory effect, with an inhibition rate of 70.98% (Figure 5A–D). This robust activity positioned J-7 as the primary candidate for subsequent broad-spectrum evaluation. The broad-spectrum antagonism of J-7 was then systematically assessed against a panel of pathogens. Subsequent evaluation against other wheat FCR pathogens revealed consistent efficacy, with inhibition rates >70% against *F. pseudograminearum*, *F. graminearum*, and *B. sorokiniana* (Figure 5E,F). Given its potent activity against FCR pathogens, we further assessed isolate J-7’s inhibitory spectrum against six additional soil-borne pathogens: *Verticillium dahliae*, *Rhizoctonia solani*, *Cytospora chrysosperma*, *Macrophomina phaseolina*, *Trichothecium roseum*, and *Valsa pyri*. Isolate J-7 maintained inhibition rates above 70% against all tested pathogens, with particularly strong activity against *V. dahliae* (91.70% inhibition) (Figure 5G,H). These results demonstrate the broad-spectrum antagonistic capability of isolate J-7, suggesting its potential as a versatile biocontrol agent.

Based on comprehensive characterization including morphological observation (Figure S4), physiological and biochemical tests (Table S10), and phylogenetic analysis of the 16S rDNA sequence (Figure 5I), isolate J-7 was identified as *Pseudomonas aeruginosa*.

### 3.7. P. aeruginosa J-7 Has Both Control and Growth-Promoting Effects

Although the in vitro antagonistic effect of J-7 was remarkable, its biocontrol efficacy against wheat crown rot was further verified via pot experiments. Three treatments were established in this study.

**Figure 6:**
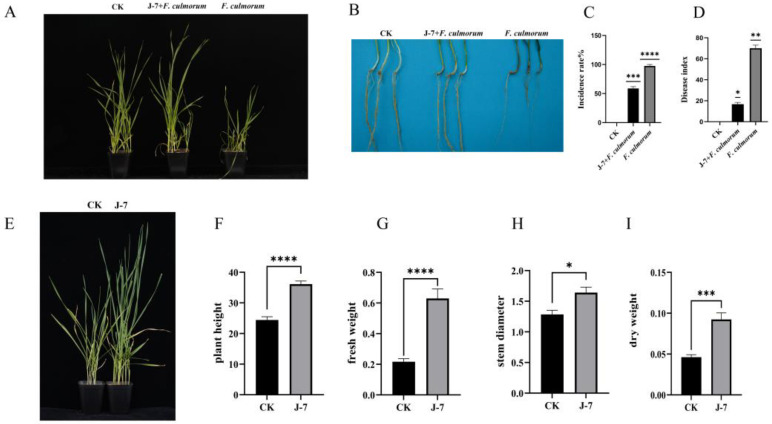
Pot experiment with *P. aeruginosa* J-7 and *F. culmorum* to verify the inhibitory effect of *P. aeruginosa* J-7 against *F. culmorum* and its growth-promoting effect on wheat. (**A**) Growth of wheat (cv. Xindong 48) 21 days after inoculation with the pathogen and *P. aeruginosa* J-7. (**B**) Disease symptoms at the wheat stem base 21 days after inoculation. (**C**) Average disease incidence of wheat 21 days after inoculation. Values represent mean ± SD of three independent experiments. (**D**) Disease index of wheat 21 days after inoculation. Values represent mean ± SD of three independent experiments. (**E**) Growth of wheat 28 days after treatment with *P. aeruginosa* J-7. (**F**–**I**) Physiological parameters of wheat (cv. Xindong 48) measured to assess the growth-promoting ability of *P. aeruginosa* J-7: (**F**) height, (**G**) fresh weight, (**H**) stem diameter, and (**I**) dry weight. Data are shown as mean ± SEM (*n* = 3 independent biological replicates). Statistical significance was determined by two-tailed Student’s *t*-test (* *p* < 0.05, ** *p* < 0.01, *** *p* < 0.001, **** *p* < 0.0001).

After 21 days of inoculation, typical FCR symptoms appeared in seedlings treated with *F. culmorum* alone, while plants inoculated with *P. aeruginosa* J-7 showed significantly reduced disease severity (Figure 6A,B). Quantitative analysis demonstrated that J-7 treatment significantly decreased both disease incidence and disease index (*p* < 0.05) (Figure 6C,D), confirming the protective effect of J-7 against wheat FCR.

The growth-promoting capacity of J-7 was also assessed. After 28 days of cultivation, seedlings treated with *P. aeruginosa* J-7 exhibited much stronger growth vigor than the control (Figure 6E). Physiological measurements showed that J-7 significantly improved plant height, fresh weight and dry weight, with dry matter accumulation more than twice that of the control (Figure 6F–I). These results demonstrate that *P. aeruginosa* J-7 can not only effectively control wheat FCR, but also promote wheat seedling growth.

### 3.8. Genomic Features of P. aeruginosa J-7 Indicates Reveal Multiple Biocontrol Mechanisms

**Figure 7:**
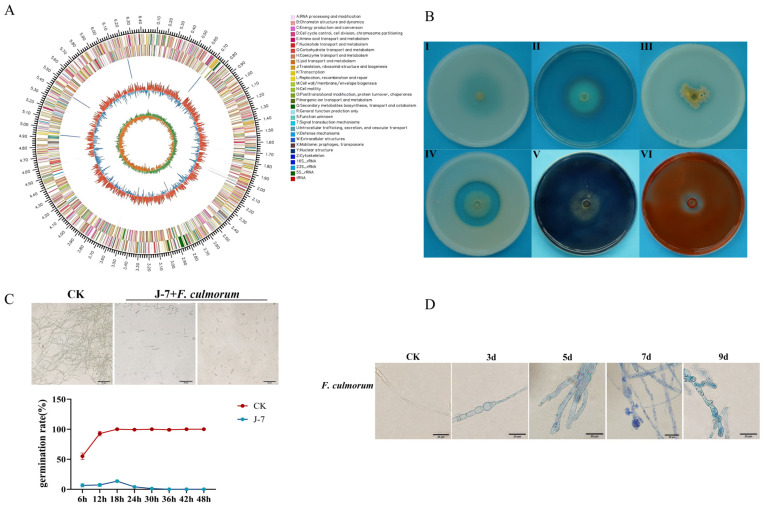
Inhibition of spore germination and mycelial damage by *P. aeruginosa* J-7. (**A**) Circular genome map of *P. aeruginosa* J-7. Notes: The outermost circle indicates genome size; the second and third circles represent CDS on positive and negative strands, with different colors indicating functional classifications of COGs; the fourth circle shows rRNA and tRNA; the fifth circle displays GC content (outward red regions indicate higher than average GC content, inward blue regions indicate lower than average); the innermost circle shows GC skew values [(G-C)/(G + C)], which assists in identifying leading/lagging strands and replication origin/terminus in circular genomes. (**B**) In vitro detection of cell wall-degrading enzymes and growth-promoting factors production by *P. aeruginosa* J-7. (**I**–**VI**): organic phosphate, siderophore, inorganic phosphate, protease, amylase, cellulase. (**C**) Inhibition of *F. culmorum* conidia germination by *P. aeruginosa* J-7 at 12 h and 24 h; line chart shows conidia germination inhibition from 6 to 24 h. While untreated spores germinated almost completely, the germination rate of XN22-1 conidia treated with the bacterial suspension started at only 6% after 6 h, reached 15% by 18 h, and was subsequently suppressed by the proliferating J-7, eventually ceasing entirely. (**D**) Microscopic observation of *F. culmorum* mycelial inhibition by *P. aeruginosa* J-7 (3–9 d). Note: Blue staining indicates trypan blue staining. Untreated hyphae appeared slender and smooth with uniform diameter, whereas the treated hyphae exhibited sequential structural alterations: initially, the internodal regions became shortened, swollen, and flattened; subsequently, the hyphal tips showed distinct swelling; further manifestations included overall shortening, vesicular swelling, and increased branching. Ultimately, the hyphae displayed progressive degenerative changes in the internal membrane structure (vacuolization), culminating in shrinkage, rupture, and cytoplasmic leakage leading to cell death.

Whole-genome sequencing of *P. aeruginosa* J-7 was performed to elucidate its genetic potential for biocontrol and plant growth promotion (Figure 7A). The complete genome comprises 6,448,507 bp with a GC content of 67.03%, encoding 4881 genes with an average length of 980.58 bp. Protein-coding sequences comprised 89.82% of the annotated genome. Genomic analysis identified 10 genes associated with biocontrol activities (Table S11). These include 20 *phz* genes involved in phenazine antibiotic biosynthesis, 9 genes (*pvdA*, *pvdQ*, *pvdG*, *pvdP*, *pvdR*, *pvdF*, *pvdN*, *pvdS*, *pvdL*) responsible for pyoverdine siderophore production, and 8 *pch* genes required for pyochelin siderophore synthesis. Additionally, the genome contains 1 *rhlR*, a key regulatory gene for rhamnolipid biosynthesis. Several plant growth-promoting genes were also identified, including phoQ involved in phosphate metabolism. These genomic features collectively indicate that *P. aeruginosa* J-7 possesses substantial potential for both biological control and plant growth promotion.

### 3.9. Antagonistic Mechanisms of P. aeruginosa J-7 Against F. culmorum

*P. aeruginosa* J-7 demonstrated the ability to produce multiple cell wall-degrading enzymes, as evidenced by clear hydrolysis zones (48.86%, 31.11%, 13.63%) on specific detection media for protease, cellulase, and amylase (Figure 7B(IV–VI)). The strain also exhibited plant growth-promoting traits, showing siderophore production and phosphate solubilization capacity through formation of characteristic zones (57.36%, 44.23%, 29.85%) on CAS and phosphorus media (Figure 7B(I–III)).

To investigate the antifungal activity against *F. culmorum*, we examined the effect of *P. aeruginosa* J-7 on conidia germination and mycelial morphology. In co-culture experiments with *P. aeruginosa* J-7 bacterial suspension (OD_600_ = 1.0), *F. culmorum* XN22-1 conidia germination was strongly inhibited. Untreated conidia showed rapid germination, exceeding 50% at 6 h and nearly reaching completion (98%) by 12 h. In contrast, the germination of *P. aeruginosa* J-7-treated conidia was drastically inhibited, plateauing at only 15% by 18 h before ceasing entirely (Figure 7C). Microscopic examination revealed that *P. aeruginosa* J-7 induced severe morphological alterations in *F. culmorum* mycelia, such as hyphal shortening, swelling, tip deformation, vesiculation, and excessive branching (Figure 7D).

Trypan blue staining revealed a time-dependent loss of membrane integrity in *P. aeruginosa* J-7-treated hyphae, accompanied by progressive cellular degeneration—including vacuolization, cytoplasmic leakage, and eventual cell death—which intensified from 3 to 9 days of exposure. Collectively, our findings point to a multi-faceted antagonistic strategy employed by *P. aeruginosa* J-7. The combined action of cell wall-degrading enzymes, siderophore-mediated competition, and direct physical damage to fungal structures effectively suppresses the growth and development of *F. culmorum*.

## 4. Discussion

In 2024, Gao et al. confirmed the occurrence of FCR caused by *F. culmorum* on winter wheat in China’s Xinjiang Uygur Autonomous Region [12]. These findings suggest a more complex pathogenic composition in Xinjiang compared to other wheat-growing regions in China, compounded by notable environmental differences between the northern and southern parts of the region. Despite these insights, the overall occurrence and distribution of FCR in Xinjiang remain poorly characterized.

As a major and persistent disease in global wheat production, Fusarium crown rot (FCR) has emerged as a significant threat to wheat safety in Xinjiang. In this study, we identified *F. culmorum* as the core causal agent of wheat FCR in Xinjiang, accounting for 73.6% of the isolates. This result fills a knowledge gap regarding the causal agents of FCR in Xinjiang. Our findings show that *F. culmorum* is the dominant pathogen in this region. This differs from reports in major wheat-growing areas such as Australia and the U.S. Pacific Northwest, where *F. pseudograminearum* is more prevalent [36,37,38,39]. Most previous studies have suggested that *F. pseudograminearum* dominates in drier and warmer regions, while *F. culmorum* is more common in cooler and wetter environments [40,41,42,43,44,45]. In China, the distribution of FCR pathogens follows clear climate-dependent patterns. On the North China Plain (including Henan, Shandong, Jiangsu, and parts of Shaanxi and Shanxi), *F. pseudograminearum* has been the dominant pathogen since 2011. This matches the warm and relatively arid climate in this region [46,47]. In wheat–rice rotation areas such as Jiangsu and Anhui, frequent rainfall during wheat heading favors *F. asiaticum* as the primary pathogen, followed by *F. graminearum*. In cooler and more humid regions such as Xingtai and Handan in Hebei Province, *F. culmorum* is the dominant species, consistent with local climatic conditions [48]. Our results clarify the pathogen composition of FCR in this important wheat-producing region. They also reveal an interesting ecological pattern: Xinjiang has an arid climate, which would theoretically favor *F. pseudograminearum*, yet *F. culmorum* is dominant here.

The dominance of *F. culmorum* in an arid environment challenges simplistic climate-pathogen distribution models. This pattern is consistent with reports from dry regions such as Algeria, where *F. culmorum* is also dominant. Several factors may explain this contradiction. First, drought tolerance in some *F. culmorum* strains may support their adaptation to Xinjiang’s dry conditions [49,50]. Second, local irrigation creates favorable rhizosphere microenvironments that alleviate regional aridity. Niche specialization also differs between species: *F. culmorum* is more aggressive to seeds and heads, whereas *F. pseudograminearum* mainly damages crown tissues. This difference helps explain the dominance of *F. culmorum* in early wheat growth stages. Similar to Algeria, the low prevalence of *F. pseudograminearum* may result from local agronomic practices, microclimates, or historical pathogen establishment. Overall, pathogen distribution is shaped not only by climate but also by ecological adaptation and agricultural management. Future work should investigate the genetic diversity and functional traits of *F. culmorum* in Xinjiang to clarify its regional dominance.

The clear identification of *F. culmorum* as the dominant pathogen underscores the urgency of developing effective control measures. Compounding this urgency, the lack of resistant wheat varieties in Xinjiang is a major concern. Our resistance screening of 30 local and introduced varieties revealed that only three exhibited moderate resistance, underscoring an urgent need for breeding programs focused on FCR resistance. This aligns with global challenges where fully resistant varieties remain scarce, and most commercial cultivars show susceptibility [51,52,53]. Mid-resistance was observed in Xinchun 19, Xinchun 50, and Youpi 23, which provide valuable genetic resources for future breeding.

In this, we turn our attention to biological control. The enrichment of *Pseudomonas* in the rhizosphere of healthy seedlings suggests a protective role of specific bacterial consortia against FCR. *P. aeruginosa* J-7, which exhibits strong broad-spectrum antagonism against multiple pathogens, including *F. culmorum*, *F. graminearum*, and *B. sorokiniana*, highlights its potential as a biocontrol agent. The efficacy of *P. aeruginosa* J-7 in reducing disease incidence and index in pot experiments supports its practical applicability, although field trials are necessary to confirm these results under natural conditions. Genomic analysis of *P. aeruginosa* J-7 revealed the presence of genes involved in the synthesis of phenazines, pyoverdine, pyochelin, and rhamnolipids—compounds known to contribute to antibiosis and induced systemic resistance [54,55,56,57]. The production of siderophores and cell wall-degrading enzymes further enhances its antagonistic capability, facilitating direct damage to fungal structures and inhibition of conidia germination. These mechanisms are consistent with those reported in other *pseudomonads* used in biocontrol. Li et al. reported that *P. aeruginosa* strain Pa608, isolated from pepper rhizosphere, inhibited *Phytophthora capsici* with 88.0% efficacy in pot trials [58]. Similarly, strain CQ-40 suppressed gray mold in tomato, and strain ZL6 controlled cotton *Verticillium* wilt. *P. aeruginosa* also shows compatibility with *Trichoderma* spp., and their combination exhibits synergistic effects against *Fusarium solani* [59,60,61,62]. These studies collectively confirm that *P. aeruginosa* possesses broad-spectrum disease-suppressive capabilities, mediated by diverse mechanisms such as siderophore production, antibiotic synthesis (e.g., phenazine derivatives), cell wall-degrading enzyme secretion, and induction of systemic resistance. Therefore, further exploration of the multi-target antifungal functions of this bacterium holds great promise for developing integrated “one-strain-against-multiple-diseases” [63] strategies, with considerable theoretical and practical significance.

In conclusion, this study not only clarifies the pathogenic landscape of wheat FCR in Xinjiang but also offers a viable biocontrol candidate in *P. aeruginosa* J-7. Integrating such microbial agents with agronomic practices and resistant varieties could form a sustainable strategy for managing FCR, reducing reliance on chemical fungicides and contributing to eco-friendly agricultural practices.

The wheat rhizosphere-isolated *P. aeruginosa* J-7 is conspecific with the rhizosphere biocontrol strain *P. aeruginosa* M18 reported by Wu et al. (2011) [64], sharing prominent antifungal potential—consistent with M18’s ability to produce phenazine-1-carboxylic acid (PCA) and pyoluteorin (Plt). This confirms J-7 as an environmental isolate, analogous to M18, and provides a basis for agricultural biosafety evaluation. Wu’s comparative analysis of M18 and clinical *P. aeruginosa* strains (e.g., PAO1, LESB58) offers direct insights into J-7’s traits. Genetically, M18 lacks clinical strain-specific pathogenic genomic islands (e.g., PAPI-1) but harbors biocontrol-enriched accessory genomes and restriction-modification (RM) systems, while clinical strains carry complete virulence/drug-resistance gene clusters. Phenotypically, M18 exhibits attenuated pathogenicity (easily cleared in mouse models), weaker biofilm formation, and lower antibiotic resistance than clinical isolates. These characteristics are inferred to be shared by J-7, reflecting rhizosphere vs. clinical niche-driven divergence.

The agricultural biocontrol superiority of J-7 (and M18) stems from rhizosphere adaptive evolution: minimal insertion sequences (IS) and abundant CRISPR elements preserve biocontrol gene clusters; RM systems restrict exogenous pathogenic gene transfer; temperature-dependent transcription upregulates biocontrol genes at 28 °C (rhizosphere temperature) without activating virulence at 37 °C; and virulence gene degradation alongside plant-beneficial pathway enrichment enhances rhizosphere competitiveness. Key application challenges include inherent biosafety risks as a conditional pathogen, field temperature-induced biocontrol gene expression fluctuations, limited long-term colonization due to weak biofilm formation, and fungal resistance risks from reliance on a few secondary metabolites.

Future work should validate J-7’s genomic/phenotypic differences from clinical strains (following Wu’s framework), optimize temperature-stabilized formulations, and conduct long-term environmental monitoring to ensure sustainable application in wheat Fusarium crown rot control.

## Data Availability

This study did not generate new unique reagents. They have shared my data at Genome Sequence Archive (GSA) BioProject No.: PRJCA048791, under the GSA accession number CRA032272, CRA032267, CRA037583. Lead contact: Requests for further information and resources should be directed to and will be fulfilled by the lead contact, Qinggui Lian (lianqg@shzu.edu.cn).

## Supplementary Materials

The following supporting information can be downloaded at: https://www.mdpi.com/article/10.3390/microorganisms14030627/s1, Figure S1: Pathogenicity identification of class II *Fusarium* and class I *Bipolaris*; Figure S2: Morphological and molecular phylogenetic analysis of class II *Fusarium* and class I *Bipolaris*; Figure S3: Microbial diversity analysis of soil samples. (A) Dilution curve (left) Bacterial community dilution curve, (right) Fungal community dilution curve; Figure S4: Culture morphology of strain J-7; Table S1: Severity classification and symptom description of wheat crown rot at seedling stage; Table S2: Polymerase chain reaction system; Table S3: Reaction procedure of PCR amplif; Table S4: All primers used in this article; Table S5: All primers used in this article; Table S6: Occurrence of Fusarium crown rot in Yumin and Xinhe County; Table S7: Preliminary classification of fungus isolates in *Fusarium* crown; Table S8: Isolating frequency statistics of three pathogens; Table S9: Resistance evaluation of 30 wheat varieties to *F. culmorum*; Table S10: Physiological and biochemical characteristics of isolate J-7; Table S11: Annotation summary of isolate J-7. Refs. [29,65] can be found in Supplementary Materials.

## References

[1] Gazza L., Nocente F. (2023). The contribution of minor cereals to sustainable diets and agro-food biodiversity. Foods.

[2] Melania F., Hammond-Kosack E., Solomon P. (2018). A review of wheat diseases-a field perspective. Mol. Plant Pathol..

[3] Neupane B., Bisek B., Marais F. (2024). A diallel study to detect genetic background variation for FHB resistance in winter wheat. Sci. Rep..

[4] Wearing A.H., Burgess L.W. (1977). The relative frequency of isolation of Fusaria from wheat soils in the eastern Australian wheat belt. Aust. Plant Pathol. Soc. Newsl..

[5] Su Y., Xu X., Wang Y., Wang T., Yu J., Yang J., Li J., Gao Y., Wang Y., Sang W. (2025). Identification of genetic loci and candidate genes underlying Fusarium crown rot resistance in wheat. Theor. Appl. Genet..

[6] Claude B., Paula B., Elisavet C., Francesco D., Paolo G. (2022). Pest categorisation of *Fusarium pseudograminearum*. EFSA J..

[7] Murray G.M., Brennan J.P. (2009). Estimating disease losses to the Australian wheat industry. Australas. Plant Pathol..

[8] Hua L., Song R., Hao X., Zhang J., Liu Y., Luo J., Ren X., Li H., Wang G., Renhman S.U. (2025). Manipulation of the brown glume and internode 1 gene leads to alterations in the colouration of lignified tissues, lignin content and pathogen resistance in wheat. Plant Biotechnol. J..

[9] Li H.L., Yuan H.X., Fu B., Xing X.P., Sun B.J., Tang W.H. (2012). First report of *Fusarium pseudograminearum* causing crown rot of wheat in Henan, China. Plant Dis..

[10] Zhang J., Zhan J., Wang J., Zhang M., Li C., Wang W., Suo Y., Song F. (2024). Population genetic analyses and trichothecene genotype profiling of *Fusarium pseudograminearum* causing wheat crown rot in Henan, China. J. Fungi.

[11] Xia Y., Yubo P., Singh P.K., He X., Ren Y., Zhao L., Zhang N., Cheng S., Chen F. (2019). Investigation and genome-wide association study for Fusarium crown rot resistance in Chinese common wheat. BMC Plant Biol..

[12] Gao Y., Li J., Li Y., Cao W., Deng F., Niu W., Shen Y., Li Y., Li G., Gao H. (2024). Occurrence of Fusarium crown rot caused by *Fusarium culmorum* on winter wheat in Xinjiang uygur autonomous region, China. Plant Dis..

[13] Yang M., Yii L.S., Li G.K., Li T.G., Zhang H., Gao H. (2024). First Report of Crown Rot Caused by *Fusarium graminearum* on Wheat in Xinjiang Uygur Autonomous Region, China. Plant Dis..

[14] Lefan P., Farhan G., Zeng M., Wu D., Zeng Q., Han D., Li C. (2022). Source identification and genome-wide association analysis of crown rot resistance in wheat. Plants.

[15] Alahmad S., Simpfendorfer S., Bentley A.R., Hickey L.T. (2018). Crown rot of wheat in Australia: *Fusarium pseudograminearum* taxonomy, population biology and disease management. Australas. Plant Pathol..

[16] Bak G.R., Lee K.K., Clark I.M., Mauchline T.H., Kavamura V.N., Jee S., Lee J.T., Kim H., Lee H. (2025). Changes in the potato rhizosphere microbiota richness and diversity occur in a growth stage-dependent manner. Sci. Rep..

[17] Sun T., Liu H., Wang N., Huang M., Banerjee S., Jousset A., Xu Y., Shen Q., Wang S., Wang X. (2025). Interactions with native microbial keystone taxa enhance the biocontrol efficiency of Streptomyces. Microbiome.

[18] Jin S., Alberti F. (2025). Advances in the discovery and study of Trichoderma natural products for biological control applications. Nat. Prod. Rep..

[19] Li P., Feng B., Sun Y., Yang Y. (2025). Biocontrol potential of rhizosphere *Streptomyces* LWT20 against postharvest decay on grape caused by Botrytis cinerea. LWT-Food Sci. Technol..

[20] Li J., Song Z., Wang Y., Chen C., Jiang H., Ding T., Xie S. (2025). Root exudates mediate *Bacillus velezensis* FZB42’s colonization-independent biocontrol in maize. J. Agric. Food Chem..

[21] Zhu M., Liu J., Zhang F., Qiu Z., Zhao S. (2025). Biocontrol potential of *Bacillus velezensis* against the postharvest pink mold rot in Citrus reticulata and investigations on some of the mechanisms of action. Postharvest Biol. Technol..

[22] Rupali G., Ravindran K., Meirav L.M., Sabina M., Dalia R., Ran S., Yigal E., Dana M., Maya B. (2024). *Bacillus thuringiensis* promotes systemic immunity in tomato, controlling pests and pathogens and promoting yield. Food Secur..

[23] Xia X., Wei Q., Wu H., Chen X., Xiao C., Ye Y., Liu C., Yu H., Guo Y., Sun Y. (2024). *Bacillus* species are core microbiota of resistant maize cultivars that induce host metabolic defense against corn stalk rot. Microbiome.

[24] Jiang N., Qiu J., Tian D., Shi H., Liu Z., Wen H., Xie S., Chen H., Wu M., Kou Y. (2025). Mixture of *Bacillus amyloliquefaciens* and *Bacillus pumilus* modulates community structures of rice rhizosphere soil to suppress rice seedling blight. Rice Sci..

[25] Zhang N., Wang Z., Shao J., Xu Z., Liu Y., Xun W., Miao Y., Shen Q., Zhang R. (2023). Biocontrol mechanisms of *Bacillus*: Improving the efficiency of green agriculture. Microb. Biotechnol..

[26] Ren C., Li S., Li P., Wang Y., Yuan H., Zhao Q., Li H., Li F., Han Y. (2025). Pathogen-activated *Chaetomium globosum* G3 enhances iron competition and other antagonistic mechanisms to suppress maize seedling blight causal agent *Fusarium verticillioides*. Microbiol. Res..

[27] O’Sullivan C.A., Roper M.M., Myers C.A., Thatcher L.F. (2021). Developing *Actinobacterial* endophytes as biocontrol products for *Fusarium pseudograminearum* in wheat. Front. Bioeng. Biotech..

[28] Liu L., Jin Y., Lian H., Yin Q., Wang H. (2024). Exploring the biocontrol potential of *Phanerochaete chrysosporium* against wheat crown ro. J. Fungi.

[29] Martin A., Simpfendorfer S., Hare R.A., Sutherland M.W. (2013). Introgression of hexaploid sources of crown rot resistance into durum wheat. Euphytica.

[30] Amelung D., Dehne H., Adam G., Diekmann M., Frahm J., Mauler-Machnik A., van Halteren P. (1997). Experiences with the Isolation of Plant Pathogenic Fungi. Diagnosis and Identification of Plant Pathogens.

[31] Mitter V., Zhang M.C., Liu C.J., Ghosh M., Chakraborty S. (2006). A high-throughput glasshouse bioassay to detect crown rot resistance in wheat germplasm. Plant Pathol..

[32] Leslie J., Summerell B. (2006). Morphological Characters. The Fusarium Laboratory Manual.

[33] Hyatt D., Chen G., LoCascio P.F., Land M., Larimer F.W., Hauser L.J. (2010). Prodigal: Prokaryotic gene recognition and translation initiation site identification. BMC Bioinform..

[34] Besemer J., Borodovsky M. (2005). GeneMark: Web software for gene finding in prokaryotes, eukaryotes and viruses. Nucleic Acids Res..

[35] Chan P.P., Lowe T.M. (1962). tRNAscan-SE: Searching for tRNA Genes in Genomic Sequences. Gene Prediction.

[36] Pu L., Jin Q., Cai X., Qu C., Zhang J., Bai X., Gao J., Kang Z., Gao J. (2025). Crown rot in wheat: Pathogen biology, host responses, and management strategies. Stress Biol..

[37] Bozoğlu T., Derviş S., Imren M., Amer M., Özdemir F., Paulitz T.C., Morgounov A., Dababat A.A., Öze.r G. (2022). Fungal pathogens associated with crown and root rot of wheat in central, eastern, and southeastern Kazakhstan. J. Fungi.

[38] Özer G., Erper İ., Yıldız Ş., Bozoğlu T., Zholdoshbekova S., Alkan M., Tekin F., Uulu T.E., İmren M., Dababat A.A. (2023). Fungal pathogens associated with crown and root rot in wheat-growing areas of northern Kyrgyzstan. J. Fungi.

[39] Zhao X., Hou D., Xu J., Wang K., Hu Z. (2022). Antagonistic activity of fungal strains against Fusarium crown rot. Plants.

[40] Xu W., Yang Q., Xie X., Goodwin P.H., Deng X., Zhang J., Sun R., Wang Q., Xia M., Wu C. (2022). Genomic and phenotypic insights into the potential of *Bacillus subtilis* YB-15 isolated from rhizosphere to biocontrol against crown rot and promote growth of wheat. Biology.

[41] Jamila B., Hugh W., Mohammed B., Mustapha L., Llyass M., Fatiha B. (2022). Evaluation of durum wheat genotypes for resistance against root rot disease caused by Moroccan *Fusarium culmorum* isolates. Plant Pathol. J..

[42] Jagdeep S., Bhavit C., Ali R., Yang S., Sandhu S. (2023). Important wheat diseases in the US and their management in the 21st century. Front. Plant Sci..

[43] Vandicke J., Visschere D.K., Croubels S., Saeger S.D., Audenaert K., Haesaert G. (2019). Mycotoxins in Flanders’fields: Occurrence and correlations with *Fusarium* species in whole-plant harvested maize. Microorganism.

[44] Birr T., Hasler M., Verreet J., Klink H. (2020). Composition and predominance of *Fusarium* species causing Fusarium head blight in winter wheat grain depending on cultivar susceptibility and meteorological factors. Microorganisms.

[45] Andrew M., Brad B., Steven S., Herdina, Daniele G.D., Yang N., Beverly O., Ben O. (2023). Improved quantification of *Fusarium pseudograminearum* (Fusarium crown rot) using qPCR measurement of infection in multi-species winter cereal experiments. Front. Plant Sci..

[46] Zhuo H., He X., Wang S., Ma Q., Sun B., Ding S., Chen L., Zhang M., Li H. (2019). Diversity of the *Fusarium* pathogens associated with crown rot in the Huanghuai wheat-growing region of China. Environ. Microbiol..

[47] Deng Y., Li W., Zhang P., Sun H., Zhang X., Zhang A., Chen H. (2020). *Fusarium pseudograminearum* as an emerging pathogen of crown rot of wheat in eastern China. Plant Pathol..

[48] Ji L., Li Q., Wang Y., Burgess L.W., Sun M., Cao K., Kong L. (2019). Monitoring of *Fusarium* species and trichothecene genotypes associated with Fusarium head blight on wheat in hebei province, China. Toxins.

[49] Abdallah-Nekache N., Laraba I., Ducos C., Barreau C., Bouznad Z., Boureghda H. (2019). Occurrence of Fusarium head blight and Fusarium crown rot in Algerian wheat: Identification of associated species and assessment of aggressiveness. Eur. J. Plant Pathol..

[50] Bennacer A., Halouane F.S., Alvarez M., Oukali Z., Bennacer N.E.H., Foughalia A., Delgado J. (2025). *Talaromyces pinophilus* strain HD25G2 as a novel biocontrol agent of *Fusarium culmorum*, the causal agent of root and crown rot of soft wheat. J. Fungi.

[51] Mahbubur R.M., Philip D., Urmil B., Raj P., Matthew H., Richard T. (2021). Relationship between resistance and tolerance of crown rot in bread wheat. Field Crop. Res..

[52] Rahman M., Davies P., Bansal U., Pasam R., Hayden M., Trethowan R. (2020). Marker-assisted recurrent selection improves the crown rot resistance of bread wheat. Mol. Breed..

[53] Martin A., Bovill W.D., Percy C.D., Herde D., Fletcher S., Kelly A., Neate S.M., Sutherland M.W. (2015). Markers for seedling and adult plant crown rot resistance in four partially resistant bread wheat sources. Theor. Appl. Genet..

[54] Hossain M.S., Sharna A.A., Sharmin S., Tusty T.A., Hashem A., Sarker P.K. (2025). Exploring the antibacterial potential of environmental *Pseudomonas aeruginosa* isolates: Insights from in vitro studies and genome mining approaches. J. Genet. Eng. Biotechnol..

[55] Vasquez-Rifo A., Ricci E.P., Ambros V. (2020). *Pseudomonas aeruginosa* cleaves the decoding center of Caenorhabditis elegans ribosomes. PLoS Biol..

[56] Haripriyan J., Binu C.R., Menon N.D., Vanuopadath M., Hari M.B., Namitha N., Binoy K., Kumar A., Nair B.G., Nizet V. (2025). Essential oils modulate virulence phenotypes in a multidrug-resistant pyomelanogenic *Pseudomonas aeruginosa* clinical isolate. Sci. Rep..

[57] Phelan V.V., Fang J., Dorrestein P.C. (2015). Mass spectrometry analysis of *Pseudomonas aeruginosa* treated with azithromycin. J. Am. Soc. Mass Spectrom..

[58] Li C., Gao X., Huo Y., Asseri T.A.Y., Tian X., Luo K. (2024). Evaluation of biocontrol efficacy of rhizosphere *Pseudomonas aeruginosa* for management of *Phytophthora capsici* of pepper. PLoS ONE.

[59] Wang X., Zhou X., Cai Z., Guo L., Chen X., Chen X., Liu J., Feng M., Qiu Y., Zhang Y. (2021). A Biocontrol Strain of *Pseudomonas aeruginosa* CQ-40 Promote Growth and Control Botrytis cinerea in Tomato. Pathogens.

[60] Jatan R., Kamboj R., Kumar M., Nitin K., Priyanka J., Charu L., Joshitha V., Vandana R., Kumar S.N., Singh B.D. (2023). Isolation and whole genome sequencing of *Pseudomonas aeruginosa* strain RK1 and its biocontrol potential against phytopathogens of rice. Biologia.

[61] Niu Q., Lei S., Zhang G., Wu G., Zhuo T., Chen K., Lin Z. (2024). Inhibition of Verticillium wilt in cotton through the application of *Pseudomonas aeruginosa* ZL6 derived from fermentation residue of kitchen waste. J. Microbiol. Biotechnol..

[62] Alemayehu D., Tesfaye A., Yitbarek W. (2021). In-vitro compatibility assay of indigenous *Trichoderma* and *Pseudomonas* species and their antagonistic activities against black root rot disease (*Fusarium solani)* of faba bean (*Vicia faba* L.). BMC Microbiol..

[63] Wang R., Wang C., Zuo B., Liang X., Zhang D., Liu R., Yang L.N., Lu B.H., Wang X., Gao J. (2021). A novel biocontrol strain *Bacillus amyloliquefaciens* FS6 for excellent control of gray mold and seedling diseases of ginseng. Plant Dis..

[64] Wu D.Q., Ye J., Ou H.Y., Wei X., Huang X., He Y.W., Xu Y. (2011). Genomic analysis and temperature-dependent transcriptome profiles of the rhizosphere originating strain *Pseudomonas aeruginosa* M18. BMC Genom..

[65] Zhuang X. (2023). Resistance Identification and Resistance Gene Analysis of Main and New Wheat Varieties to Stem Base Rot in Huanghuai Wheat Area.

