# Supplementary material for: Identification of Pathogens and Biological Control of Wheat Fusarium Crown Rot in Xinjiang with Pseudomonas aeruginosa J-7
Source: Microorganisms. 2026 Mar 10;14(3):627. doi: 10.3390/microorganisms14030627 (PMC13028721; doi:10.3390/microorganisms14030627)
Supplement: Supplementary file 1 [file microorganisms-14-00627-s001.zip › microorganisms-4091630-supplementary.pdf]

Supplementary Table S1. Severity classification and symptom description of wheat crown rot at seedling stage

disease grades	symptoms described
0	No obvious symptoms
1	There were obvious necrotic spots on the coleoptile or the first leaf sheath
2	Partial necrosis of the first leaf sheath and subcoronal segment
3	The second leaf sheath and subcoronal segment were completely necrotic with clear shortening, which did not exceed the plant or seedling
4	Part or complete necrosis of the third leaf or leaf sheath and subcoronal segment, accompanied by severe shortening overplant
5	or seedling height of the whole plant severely necrotic to completely necrotic

Note: The disease index was calculated by referring to the disease classification method of Fan Xuefeng[1] wheat stem base rot seedling stage.

Supplementary Table S2. Polymerase chain reaction system

**Teagent**	**Volume（μL）**
DNA template	1.0
2×TaqMix	5.0
Upstream primer	0.3
Downstream primer	0.3
DdH_2O	3.4
Bulk volume	10.0

Supplementary Table S3. Reaction procedure of PCR amplif

**Primer**	**Pre-denaturation**	**Degeneration**	**Annealing**	**Elongation**	**Final extension**	**Recurring number**
ITS1/ITS4	94℃，5 min	94℃，40 s	55℃，40 s	72℃，45s	72℃，10min	35
EF1-728F/EF1-986R	94℃，5min	95℃，1min	55℃，1min	72℃，30s	72℃，10min	35

Supplementary Table S4. All primers used in this article

**Primer**	**Primer sequence（5’-3’）**	**Amplified regions**
ITS1	TCCGTAGGTGAACCTGCGG	ITS
ITS4	TCCTCCGCTTATTGATATGC	
EF1-728F	CATCGAGAAGTTCGAGAAGG	*tef1-α*
EF1-986R	TACTTGAAGGAACCCTTACC	
MAT1-1F	CCTTATGACTCCCCCGATGTCG	MAT1-1
MAT1-1R	TGTTTACCACCAGACCGGAC	
MAT1-2F	AAGCGCGCTCATGTCAGTCCAAGTT	MAT1-2
MAT1-2R	GCCCACCGACAGTATTCCTT	

Supplementary Table S5. Evaluation of disease resistance grade of wheat varieties

**Disease resistance grade**	**Disease index**
Highly susceptible (HS):	$40 < DI \leq 100$
Susceptible (S)	$30 < DI \leq 40$
Moderately resistant (MR)	$20 < DI \leq 30$
Resistant (R)	$10 < DI \leq 20$
Highly resistant (HR)	$0 < DI \leq 10$
Immune (I)	$DI = 0$

Note: According to the average disease index, the resistance of 30 wheat varieties was evaluated by referring to the stem base rot resistance of Zhuangxunyu[2] wheat varieties.

Supplementary Table S6. Occurrence of *Fusarium* crown rot in Yumin and Xinhe County

Area		Seedling stage		Adult growth stages	
		**Incidence rate%**	**Disease index**	**Incidence rate%**	**Disease index**
Yumin county	①	30.0±11.1	12.0±4.7	56.0±8.9	38.8±8.7
	②	20.0±7.2	2.0±1.6	28.0±18.6	24.8±19.1
	③	15.0±13.3	4.0±14.8	21.0±22.6	11.8±23.0
	④	3.0±16.8	0.6±18.4	10.0±21.9	5.6±23.0
	⑤	5.0±15.6	1.0±17.0	8.0±13.3	3.4±22.0
	average	14.6	3.9	24.6	16.9
Xinhe county	①	36.6±1.5	35.2±5.4	54.3±4.5	50.4±5.9
	②	37.8±1.4	35.8±4.5	53.2±4.3	48.8±5.5
	③	38.0±1.4	27.6±2.7	57.0±5.0	48.8±5.9
	④	35.0±0.8	23.3±3.3	52.5±4.3	48.2±6.5
	⑤	35.0±1.1	27.6±1.7	45.0±5.2	36.0±7.3
	average	36.5	30.0	52.4	46.4

Supplementary Table S7. Preliminary classification of fungus isolates in Fusarium crown rot

Area	Growth period	Plant numbers	*Fusarium*		*Bipolaris*		*Alternaria*	
			**Number**	**Proportion (%)**	**Number**	**Proportion (%)**	**Number**	**Proportion (%)**
Yumin county	Seedling stage	32	5	15.6	27	84.4	0	0.0
	Adult growth stages	60	118	91.5	11	8.5	0	0.0
Xinhe county	Seedling stage	39	54	100.0	0	0	0	0.0
	Adult growth stages	65	71	87.7	5	6.2	5	6.2
aggregate		195	248	83.8	43	14.5	5	1.7

Supplementary Table S8. Isolating frequency statistics of three pathogens

Growth period	*F. culmorum*	*F. pseudograminearum*	*B. sorokiniana*
	Number	Number	Number
Seedling stage	39	0	9
Adult growth stages	39	15	4
aggregate	78	15	13
Proportion（%）	73.6	14.1	12.3

Supplementary Table S9. Resistance evaluation of 30 wheat varieties to *F. culmorum* XN22-1

**Varieties**	**Incidence rate%**	**Disease index**	**Resistance grade**
xinchun19*	43.75±1.31	22.50±0.94	MR
xinchun50*	43.75±2.14	25.00±2.96	MR
youpi23	56.25±2.76	30.00±0.93	MR
huachangmai26	68.75±3.44	40.00±3.20	S
nanda2419	62.50±2.12	41.25±1.50	HS
baidatou	75.00±4.40	42.50±2.33	HS
zhongyan196	56.25±2.77	47.50±2.50	HS
shuangshoumai6hao	87.50±2.51	51.88±1.09	HS
hemai11	87.50±3.09	51.88±3.32	HS
xindong55	87.50±3.46	53.75±3.58	HS
xindong32	81.25±2.93	55.00±3.21	HS
xindong52	81.25±3.30	55.63±3.31	HS
hemai31	93.75±3.75	57.50±2.21	HS
Fielder*	68.75±3.33	60.00±2.95	HS
ningchun16*	81.13±3.33	60.50±5.32	HS
jinlishenhua608	75.00±3.90	62.50±4.41	HS
heizhiF3-4	90.63±4.41	64.38±4.56	HS
zhenmai0926	90.00±5.50	66.00±4.45	HS
xindong22	87.50±4.12	66.25±2.68	HS
mengmai188	81.25±2.21	66.25±3.37	HS
xindong18	100.00±0.00	70.66±6.81	HS
xindong36	100.00±0.00	71.25±9.05	HS
fusui3hao	100.00±0.00	72.50±3.44	HS
hemai32	93.75±3.30	73.13±7.71	HS
yuyuan7hao	100.00±0.00	76.25±6.09	HS
shidong0358	93.75±5.51	78.75±3.21	HS
mingxian169	100.00±0.00	86.25±3.53	HS
hemai35	100.00±0.00	90.00±3.89	HS
bainong8399	100.00±0.00	95.00±3.56	HS
luomai38	100.00±0.00	98.75±1.01	HS

Note:* represents spring wheat varieties, the rest for winter wheat varieties， same below.These included eight varieties commonly used in Xinjiang production (e.g., Xindong 48, Xindong 36, Xindong 52), which were purchased from local agricultural markets; two varieties (Xindong 55 and Xindong 22) provided by the Xinhe County Agriculture and Rural Affairs Bureau; and twenty varieties widely cultivated outside Xinjiang, which were kindly supplied by Professor Lian Qinggui.

Supplementary Table S10. Physiological and biochemical characteristics of isolate J-7

**Pilot project**	**Appraisal result**
Pyocyanin production test	+
Gelatin of liquefaction test	+
Nitrate gas production test	+
amylohydrolysis	-
Growth at 42 °C	+
Oxidase test	+

Note : ' + ' is positive, ' - ' is negative.

Supplementary Table S11. Annotation summary of isolate J-7

Gene ID	Gene Name	Gene Description	Sample Name
gene0736	*phzA_B*	MULTISPECIES: phenazine biosynthesis protein PhzA	J_7
gene3176	*phzA_B*	MULTISPECIES: phenazine biosynthesis protein PhzA	J_7
gene3175	*phzA_B*	MULTISPECIES: phenazine biosynthesis protein PhzB	J_7
gene0735	*phzA_B*	MULTISPECIES: phenazine biosynthesis protein PhzB	J_7
gene0734	*aroF*	MULTISPECIES: phenazine biosynthesis protein PhzC	J_7
gene3174	*aroF*	MULTISPECIES: phenazine biosynthesis protein PhzC	J_7
gene0733	*phzD*	MULTISPECIES: phenazine biosynthesis protein PhzD	J_7
gene3173	*phzD*	MULTISPECIES: phenazine biosynthesis protein PhzD	J_7
gene0732	*phzE*	MULTISPECIES: phenazine biosynthesis protein PhzE	J_7
gene3172	*phzE*	phenazine biosynthesis protein PhzE	J_7
gene0731	*phzF*	MULTISPECIES: phenazine biosynthesis protein PhzF	J_7
gene3171	*phzF*	MULTISPECIES: phenazine biosynthesis protein PhzF	J_7
gene2295	*phzF*	MULTISPECIES: PhzF family phenazine biosynthesis protein	J_7
gene3735	*phzF*	MULTISPECIES: PhzF family phenazine biosynthesis protein	J_7
gene5058	*phzF*	MULTISPECIES: PhzF family phenazine biosynthesis protein	J_7
gene0730	*phzG*	MULTISPECIES: phenazine biosynthesis FMN-dependent oxidase PhzG	J_7
gene3170	*phzG*	phenazine biosynthesis FMN-dependent oxidase PhzG	J_7
gene0729	*phzS*	MULTISPECIES: pyocyanin biosynthetic protein PhzM	J_7
gene0737	*phzM*	MULTISPECIES: phenazine-1-carboxylate N-methyltransferase PhzM	J_7
gene1374	*RUFY1_2*	MULTISPECIES: PhzF family phenazine biosynthesis protein	J_7
gene2659	*bacA*	pyoverdine non-ribosomal peptide synthetase/polyketide synthase PvdL	J_7
gene2693	-	protein PvdP	J_7
gene2658	*pchC*	MULTISPECIES: pyoverdine biosynthesis thioesterase PvdG	J_7
gene2699	*pvdA*	MULTISPECIES: L-ornithine N(5)-monooxygenase PvdA	J_7
gene2696	*macA*	MULTISPECIES: pyoverdine export/recycling transporter periplasmic adaptor subunit PvdR	J_7
gene2689	*purN*	MULTISPECIES: pyoverdine biosynthesis hydroxyornithine transformylase PvdF	J_7
gene2691	*cefD*	MULTISPECIES: pyoverdine-tailoring periplasmic protein PvdN	J_7
gene2657	*rpoE*	MULTISPECIES: pyoverdine signaling pathway sigma factor PvdS	J_7
gene2700	*pvdQ*	MULTISPECIES: acyl-homoserine lactone acylase PvdQ	J_7

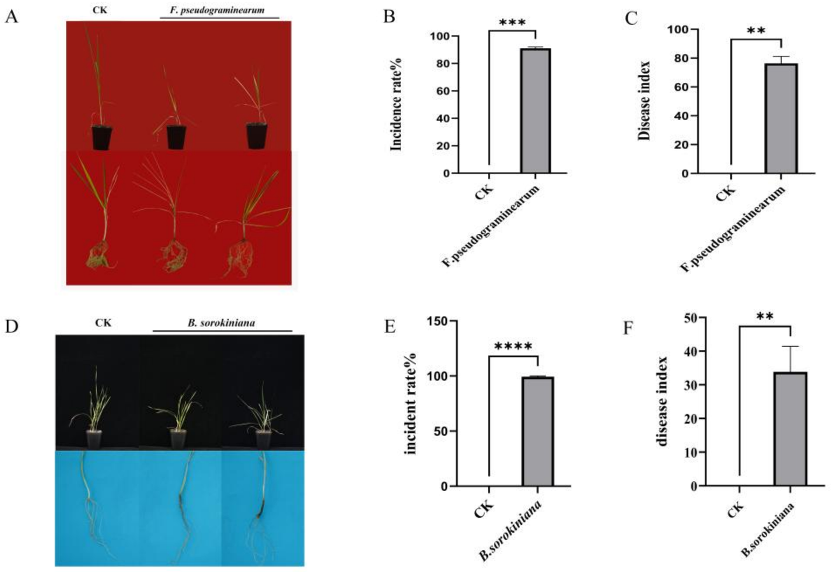

Supplement Figure S1. Pathogenicity identification of class II *Fusarium* and class I *Bipolaris* (A) The growth and incidence of wheat (Xindong 18) four weeks after inoculation of two representative strains of class II *Fusarium*. (B) The average incidence of wheat after 4 weeks of inoculation. (C) The disease index of wheat after 4 weeks. (D) The growth and incidence of wheat (Xindong 18) four weeks after inoculation with the representative strains of two class I Bipolaris. (E) The average incidence of wheat four weeks after inoculation. (F) The disease index of wheat after 4 weeks of inoculation. Data are shown as mean ± SEM (n = 5 independent biological replicates). Statistical significance was determined by two-tailed Student's t-test (*P < 0.05, **P < 0.01, ***P < 0.001).

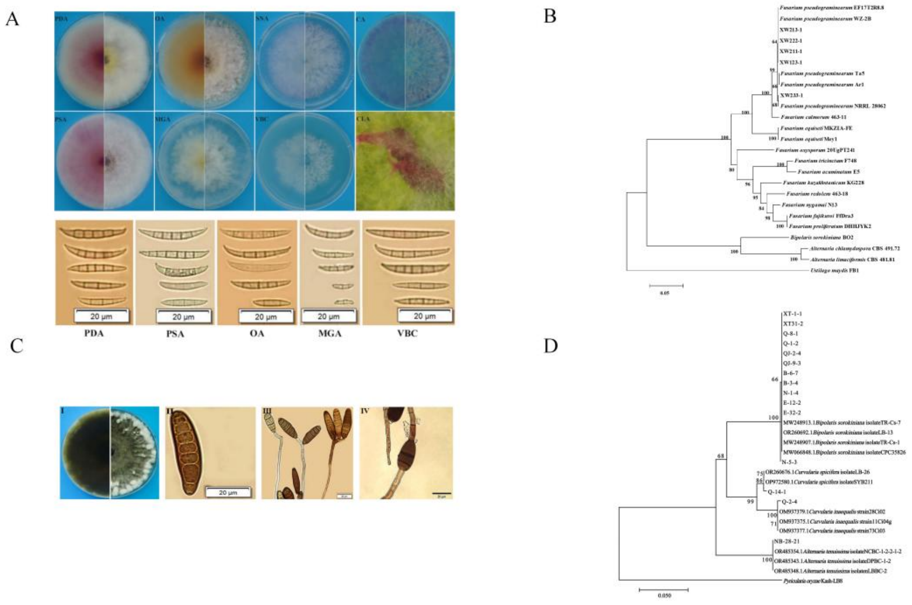

Supplement Figure S2. Morphological and molecular phylogenetic analysis of class II *Fusarium* and class I *Bipolaris*. (A) The culture characteristics and conidial morphology of the representative strain XW213-1 of class II Fusarium on 8 different media. (B) Phylogenetic tree of class II *Fusarium* based on *tef1-α* gene and ITS region nucleotide sequence. (C) The culture characteristics and conidial morphology of the representative strain XT1-1 of class I Bipolaris. Note : I PDA culture on the positive and negative side of the colony morphology, II conidia, III conidia pedicel, IV conidia germination at both ends. (D) The maximum parsimony phylogenetic tree of *Bipolaris* based on ITS-GDPH sequence.

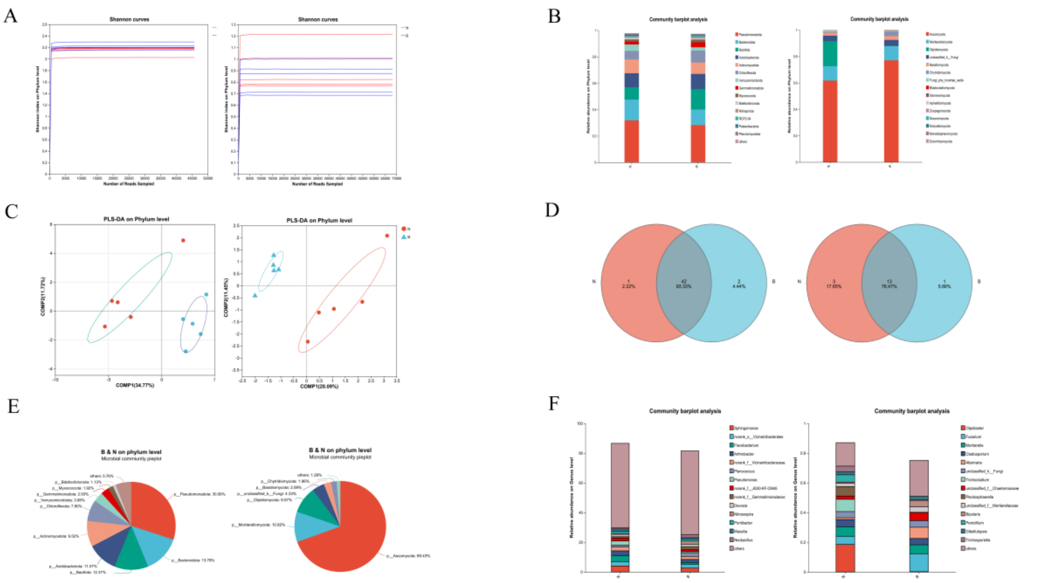

Supplement Figure S3. Microbial diversity analysis of soil samples. (A) Dilution curve (left) Bacterial community dilution curve, (right) Fungal community dilution curve. (B) Phylum level species abundance distribution histogram (left) relative abundance of bacterial community, (right) relative abundance of fungal community. (C) PLS-DA analysis of microbial community (left) PLS-DA analysis of bacteria, (right) PLS-DA analysis of fungi. (D) OTU-Venn diagram of community level characteristic number (left) bacterial OTU, (right) fungal OTU. (E) The abundance distribution of each species under the results of partial Venn analysis was pie chart (left) bacteria and (right) fungi. (F) The relative abundance of bacterial community (left) and the relative abundance of fungal community (right) in the histogram of species abundance distribution at genus level.

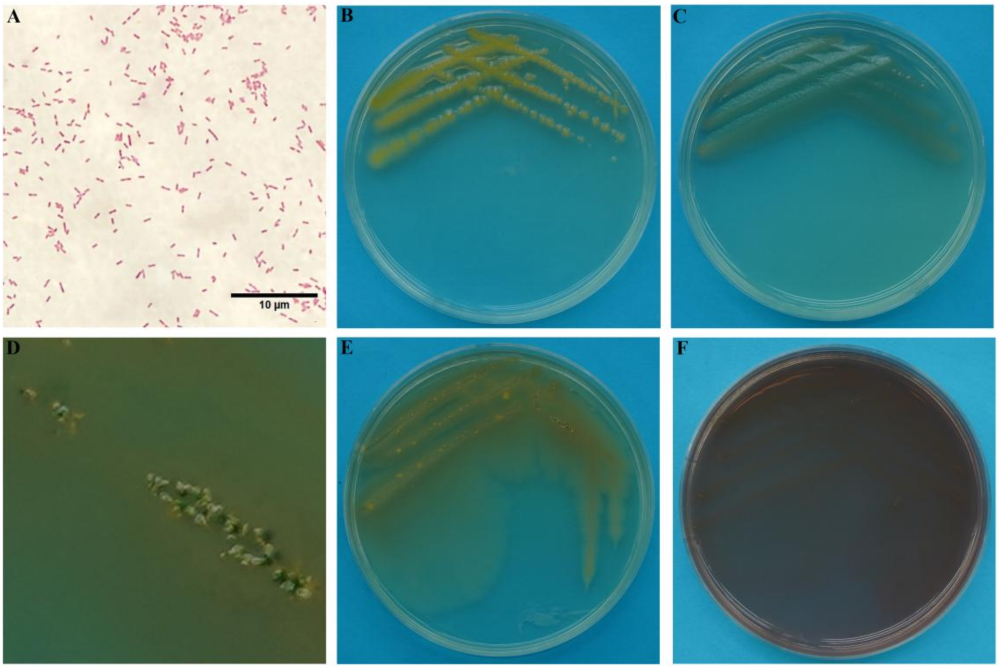

Supplement Figure S4. Culture morphology of strain J-7. (A) Gram staining of J-7 (B) and (E) colony morphology of J-7 at early and late stages of culture at 25 °C on LB (C) and (F) colony morphology of J-7 at early and late stages of culture at 37 °C on LB (D) *Pseudomonas aeruginosa* produced by the amplified strain J-7 in the figure (E).

All the media used in this article

PDA Medium: Dice 200 g of peeled potatoes and boil until thoroughly cooked. Filter the mixture, and add a solution of 15 g glucose previously dissolved in distilled water to the filtrate. Adjust the volume, set the pH to approximately 7, and distribute into 1000 mL containers pre-filled with 15 g of agar. Sterilize at 113 °C for 20 minutes.

PSA medium: The preparation method was the same as above, and glucose was replaced by sucrose.

CA Medium: Prepare following the same procedure as PDA medium, replacing peeled potatoes with carrots.

MGA Medium: Dissolve 13.40 g of yeast nitrogen base without amino acids and 10 mL of glycerol in distilled water, adjust the volume to 800 mL, add 15.00 g of agar, and mix well. Sterilize at 121 °C for 20 min. Prior to pouring plates, melt the medium and as it begins to solidify, aseptically add a filter-sterilized solution containing 0.00001 g of biotin and 0.02 g of adenine dissolved in 200 mL of sterile water.

SNA Medium: Add the following to distilled water: KH_2PO_4 1.0 g, KNO_3 1.0 g, $MgSO_4{\cdot}7H_2O$ 0.5 g, KCl 0.5 g, glucose 0.2 g, sucrose 0.2 g, and agar 15.0 g. Adjust the volume to 1000 mL and sterilize at 113 °C for 20 min.

OA Medium: Boil 30 g of oatmeal in a mesh bag for 15 min. Filter the broth, add 15 g of agar, and sterilize at 113 °C for 20 min.

VBC Medium: Add the following to distilled water: KH_2PO_4 1 g, KNO_3 1 g, sucrose 0.50 g, one compound vitamin B tablet (containing vitamin B_1 3 mg, vitamin B_2 1.5 mg, vitamin B_6 0.2 mg, nicotinamide 10 mg, calcium pantothenate 2 mg), vitamin C 0.1 g, and agar 15 g. Adjust the volume to 1000 mL and sterilize at 113 °C for 20 min.

CLA Medium: Prepare water agar by adding 15 g of agar to 1000 mL of distilled water and sterilize. When ready to use, melt the agar, surface-sterilize carnation leaves, cut them into 1.5 cm segments, and aseptically place them onto the medium just before solidification, ensuring the leaf pieces remain suspended at the surface.

CMC Medium: Add the following to 100 mL of distilled water: carboxymethyl cellulose sodium 2 g, yeast extract 0.5 g, peptone 1 g, NaCl 1 g, and agar 1.5 g. Adjust volume to 100 mL and sterilize at 113 °C for 20 minutes.

Chrome Azurol (CAS) blue dye solution: 60 mg CAS (Chrome Azurol-Z) was dissolved in 50 ml double distilled water. Solution I: Take 2.7 mg FeCl; 6H0 was dissolved in 10 m 10 m molL HCl. Solution III: 73 mg cetyltrimethylammonium bromide (HDTMA) in 40 mL double distilled water. 9 mL of solution I was dissolved in solution I, and then mixed with solution III at 121 °C for 30 min.

Organic Phosphorus Medium: Add the following to 500 mL of distilled water: glucose 5 g, $(NH_4)_2SO_4$ 0.25 g, $MgSO_4{\cdot}7H_2O$ 0.15 g, NaCl 0.15 g, KCl 0.15 g, $FeSO_4{\cdot}7H_2O$ 0.018 g, $MnSO_4{\cdot}H_2O$ 0.015 g, $CaCO_3$ 5.0 g, lecithin 0.1 g, and agar 7.5 g. Mix thoroughly, sterilize at

121 °C for 15 minutes, and pour into plates.

Inorganic Phosphorus Medium (Pikovskaya's Medium): Add the following to 500 mL of distilled water: glucose 5 g, $(NH_4)_2SO_4$ 0.25 g, $MgSO_4 \cdot 7H_2O$ 0.15 g, NaCl 0.15 g, KCl 0.15 g, $FeSO_4 \cdot 7H_2O$ 0.015 g, $MnSO_4 \cdot H_2O$ 0.015 g, $Ca_3(PO_4)_2$ 2.5 g, and agar 7.5 g. Mix thoroughly, sterilize at 121°C for 15 minutes, and pour into plates.

CMC Medium: The preparation method is the same as above.

Starch Agar Medium: Add the following to distilled water: soluble starch 20 g, peptone 10 g, beef extract 10 g, NaCl 5 g, and agar 20 g. Adjust the volume to 1000 mL and sterilize at 113 °C for 20 minutes.

Skim Milk Agar Medium: 500 mL Skim Milk, 15 g Agar

REFERENCES

[1] Martin, A.; Simpfendorfe,S. Introgression of hexaploid sources of crown rot resistance into durum wheat. *Euphytica*. 2013,192(3):463-470.

[2] Zhuang, X. Resistance identification and resistance gene analysis of main and new wheat varieties to stem base rot in Huanghuai wheat area. *Henan agricultural university*. 2023.（in Chinese）
